# Optical estimation of absolute membrane potential using fluorescence lifetime imaging

**DOI:** 10.7554/eLife.44522

**Published:** 2019-09-23

**Authors:** Julia R Lazzari-Dean, Anneliese MM Gest, Evan W Miller

**Affiliations:** 1 Department of ChemistryUniversity of California, BerkeleyBerkeleyUnited States; 2 Department of Molecular & Cell BiologyUniversity of California, BerkeleyBerkeleyUnited States; 3 Helen Wills Neuroscience InstituteUniversity of California, BerkeleyBerkeleyUnited States; YaleUnited States; The University of Texas at AustinUnited States

**Keywords:** membrane potential, cellular physiology, fluorescent indicators, Human, Other

## Abstract

All cells maintain ionic gradients across their plasma membranes, producing transmembrane potentials (V_mem_). Mounting evidence suggests a relationship between resting V_mem_ and the physiology of non-excitable cells with implications in diverse areas, including cancer, cellular differentiation, and body patterning. A lack of non-invasive methods to record absolute V_mem_ limits our understanding of this fundamental signal. To address this need, we developed a fluorescence lifetime-based approach (VF-FLIM) to visualize and optically quantify V_mem_ with single-cell resolution in mammalian cell culture. Using VF-FLIM, we report V_mem_ distributions over thousands of cells, a 100-fold improvement relative to electrophysiological approaches. In human carcinoma cells, we visualize the voltage response to growth factor stimulation, stably recording a 10–15 mV hyperpolarization over minutes. Using pharmacological inhibitors, we identify the source of the hyperpolarization as the Ca^2+^-activated K^+^ channel K_Ca_3.1. The ability to optically quantify absolute V_mem_ with cellular resolution will allow a re-examination of its signaling roles.

## eLife digest

All living cells are like tiny batteries. As long as a cell is alive, it actively maintains a difference in electrical charge between its interior and exterior. This charge difference, or voltage, is called the membrane potential, and it is vital for our bodies to work properly. For example, fast changes in membrane potential control our heartbeat and underpin the electrical signals that brain cells use to communicate.

Slower changes in membrane potential – ranging from minutes to days – may also play important roles in other organs. To understand how and why membrane potential is important in these contexts, we need methods to measure it accurately in individual cells.

One way is to puncture cells with microscopic electrodes: this yields accurate results but damages the cells and can only measure one cell at a time. Alternative methods treat cells with special fluorescent dyes and then image them with a microscope. The dyes emit light in response to voltage variations: when the cells’ membrane potential changes, the dyes glow brighter. The changes in light intensity give an estimate of the size of the change in membrane potential. This allows many cells to be analyzed without harming them, but it is less accurate.

Fluorescence lifetime refers to how long fluorescent dyes take to finish emitting light, and this phenomenon has already helped researchers to record a variety of processes in the cell. Lazzari-Dean et al. therefore wanted to use fluorescence lifetime to develop a better way of recording membrane potential. This method, called VF-FLIM, relied on measuring how long certain dyes took to finish emitting light at specific voltages, rather than how bright they were.

Experiments using mammalian cells grown in the laboratory showed that the membrane potentials measured with VF-FLIM were similar to those recorded with electrodes, which represent the highest standard of accuracy. The new method was at least eight times more accurate than other techniques using fluorescent dyes. VF-FLIM could also measure many thousands of cells within a few hours, a hundred times faster than electrode-based methods. Finally, tests on human cancer cells revealed that VF-FLIM could detect that these cells go through gradual changes in membrane potential in response to growth signals.

VF-FLIM is a new, non-invasive tool that can measure changes in membrane potential more quickly and accurately. This will help to better understand the many roles membrane potential could play in healthy and diseased cells.

## Introduction

Membrane potential (V_mem_) is an essential facet of cellular physiology. In electrically excitable cells, such as neurons and cardiomyocytes, voltage-gated ion channels enable rapid changes in membrane potential. These fast membrane potential changes, on the order of milliseconds to seconds, trigger release of neurotransmitters in neurons or contraction in myocytes. The resting membrane potentials of these cells, which change over longer timescales, affect their excitability. In non-electrically excitable cells, slower changes in V_mem_—on the order of seconds to hours—are linked to a variety of fundamental cellular processes (Abdul Kadir et al., 2018), including mitosis (Cone and Cone, 1976), cell cycle progression (Huang and Jan, 2014), and differentiation (Tsuchiya and Okada, 1982). Mounting lines of evidence point to the importance of electrochemical gradients in development, body patterning, and regeneration (Levin, 2014).

Despite the importance of membrane potential to diverse processes over a range of time scales, the existing methods for recording V_mem_ are inadequate for characterizing distributions of V_mem_ states in a sample or studying gradual shifts in resting membrane potential (Figure 1—source data 1). Patch clamp electrophysiology remains the gold standard for recording cellular electrical parameters, but it is low throughput, highly invasive, and difficult to implement over extended time periods. Where reduced invasiveness or higher throughput analyses of V_mem_ are required, optical methods for detecting events involving V_mem_ changes (e.g. whether an action potential occurred) are often employed (Huang et al., 2006; McKeithan et al., 2017; Zhang et al., 2016). However, optical approaches generally use fluorescence intensity values as a readout, which cannot report either the value of V_mem_ in millivolts (‘absolute V_mem_’) or the millivolt amount by which V_mem_ changed (Peterka et al., 2011). Variations in dye environment (Ross and Reichardt, 1979), dye loading, illumination intensity, fluorophore bleaching, and/or cellular morphology complicate fluorescence intensity measurements, making calibration and determination of absolute membrane potential difficult or impossible. This limitation restricts optical analysis to detection of acute V_mem_ changes, which can be analyzed without comparisons of V_mem_ between cells or over long timescales.

One strategy to address these fluorescence intensity artifacts and quantify cellular parameters optically is ratio-based imaging. For V_mem_ specifically, ratio-based signals can be accessed either with a two-component system or with an electrochromic voltage sensitive dye, but neither strategy has enabled accurate absolute V_mem_ recordings. Two-component FRET-oxonol systems, with independent chromophores for ratio-based calibration, have seen limited success (González and Tsien, 1997), and they confer significant capacitive load on the cell (Briggman et al., 2010). Further, their performance hinges on carefully tuned loading procedures of multiple lipophilic indicators (Adams and Levin, 2012), which can be challenging to reproduce across different samples and days. On the other hand, electrochromic probes report voltage as changes in excitation and emission wavelengths of a single chromophore (Loew et al., 1979). While they benefit from simpler loading procedures, signals from electrochromic styryl dyes require normalization with an electrode on each cell of interest to determine absolute V_mem_ accurately (Montana et al., 1989; Zhang et al., 1998; Bullen and Saggau, 1999). As a result, ratiometric V_mem_ sensors cannot be used to optically quantify slow signals in the resting V_mem_, which may be on the order of tens of millivolts. Indeed, ratiometric V_mem_ probes are most commonly applied to detect - rather than quantify - fast changes in V_mem_ (Zhang et al., 1998), much like their single wavelength counterparts.

An alternative approach to improved quantification in optical measurements is fluorescence lifetime (τ_fl_) imaging (FLIM), which measures the excited state lifetime of a population of fluorophores. Because fluorescence lifetime is an intrinsic property, FLIM can avoid many of the artifacts that confound extrinsic fluorescence intensity measurements, such as uneven dye loading, fluorophore bleaching, variations in illumination intensity, and detector sensitivity (Berezin and Achilefu, 2010; Yellen and Mongeon, 2015). If a fluorescent probe responds to the analyte of interest via changes in the lifetime of its excited state, there is the opportunity to use fluorescence lifetime to provide a more quantitative estimate of analyte parameters than can be achieved with fluorescence intensity alone. Although FLIM measurements can be affected by environmental factors such as temperature, ionic strength and local environment (Berezin and Achilefu, 2010), FLIM has been widely employed to record a number of biochemical and biophysical parameters, including intracellular Ca^2+^ concentration (Zheng et al., 2015), viscosity (Levitt et al., 2009), GTPase activity (Harvey et al., 2008), kinase activity (Lee et al., 2009), and redox state (NADH/NAD^+^ ratio) (Blacker and Duchen, 2016), among others (Yellen and Mongeon, 2015). Attempts to record absolute voltage with FLIM, however, have been limited in success (Dumas and Stoltz, 2005; Hou et al., 2014; Brinks et al., 2015). Previous work focused on genetically encoded voltage indicators (GEVIs), which either possess complex relationships between τ_fl_ and voltage (Hou et al., 2014) or show low sensitivity to voltage in lifetime (Brinks et al., 2015) and require complex and technically challenging measurements of fast photochemical kinetics to estimate voltage (Hou et al., 2014). Because of this poor voltage resolution, the fluorescence lifetimes of GEVIs cannot be used to detect most biologically relevant voltage changes, which are on the order of tens of millivolts.

**Figure 1.**
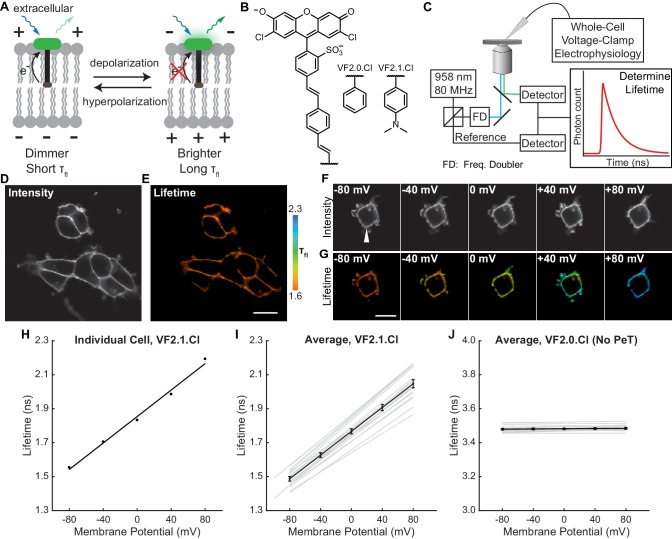
VoltageFluor FLIM linearly reports absolute membrane potential. (**A**) Mechanism of VoltageFluor dyes, in which depolarization of the membrane potential attenuates the rate of photoinduced electron transfer. (**B**) Structures of the VF molecules used in this study. (**C**) Schematic of the TCSPC system used to measure fluorescence lifetime. Simultaneous electrophysiology was used to establish lifetime-voltage relationships. (**D**) Fluorescence intensity and (**E**) lifetime of HEK293T cells loaded with 100 nM VF2.1.Cl. (**F**) Intensity and (**G**) lifetime images of HEK293T cells voltage clamped at the indicated membrane potential. (**H**) Quantification of the single trial shown in (**G**), with a linear fit to the data. (**I**) Evaluation of VF2.1.Cl lifetime-voltage relationships in many individual HEK293T cells. Gray lines represent linear fits on individual cells. Black line is the average lifetime-voltage relationship across all cells (n = 17). (**J**) VF2.0.Cl lifetime does not exhibit voltage-dependent changes. Gray lines represent linear fits on individual cells, and the black line is the average lifetime-voltage relationship across all cells (n = 17). Scale bars represent 20 μm. Error bars represent mean ± SEM. 10.7554/eLife.44522.008Figure 1—source data 1.Comparison of available approaches for measuring membrane potential in cells.^a^Measurements vary too much to be converted to absolute voltage or interpreted across populations of cells. This variability is attributable to numerous confounding factors, including dye loading, photobleaching, and sample movement (Peterka et al., 2011). ^b^While in principle less variable than a single-color fluorescence intensity measurement, in practice, the signal depends strongly on the loading of two independent lipophilic indicators (Adams and Levin, 2012; Maher et al., 2007), which can vary substantially. ^c^ANEPPS excitation ratios depend on a variety of non-voltage factors, in particular the membrane composition, leading to substantial artifacts in optical V_mem_ determinations (Zhang et al., 1998; Gross et al., 1994). ^d^With the GEVI CAESR in our hands, apparently poor protein trafficking produces large amounts of non-voltage-sensitive signal, which contaminates the FLIM recording and contributes to high cell to cell variability (Figure 1—figure supplement 4, Materials and methods). ^e^Patch-clamp electrophysiology requires physical contact with the cell of interest, which causes damage to the cell and, in whole cell configurations, washout of intracellular factors. Slight movement of the cell or sample generally result in loss of the patch. ^f^Movement of the cell and photobleaching of the dye both cause large changes to the signal over seconds to minutes. ^g^Ratio-calibrated imaging approaches use a second signal (usually another color of fluorescence) to correct for differences in dye concentration or changes in the region of interest that contaminate single-color intensity signals. If the rate of photobleaching is the same for both components, photobleaching artifacts can also be avoided. ^h^Limited by photon count rates. ^i^Limited by probe movement in the membrane, which depends mostly on lipophilicity (Briggman et al., 2010). ^j^Photon counting based lifetime imaging, like epifluorescence intensity imaging, is limited by photon count rates. Large numbers of photons per pixel must be collected to fit TCSPC FLIM data, often using a line scanning confocal approach, leading to slower acquisition speeds than epifluorescence-based intensity imaging. ^k^Toxicity from capacitive load of the sensor (Briggman et al., 2010). ^l^The spatial resolution of electrophysiology is compromised by space clamp error, preventing interpretation of V_mem_ in regions far from the electrode (e.g. many neuronal processes) (Williams and Mitchell, 2008; 35,36). ^m^As demonstrated by Cohen and co-workers (Brinks et al., 2015); in our hands with CAESR, we also experienced significant improvements in voltage resolution by fitting a single curve per FLIM image instead of processing the images pixel-wise (see Materials and methods) ^n^In this work, we calibrated VF-FLIM for V_mem_ measurements with single cell resolution. In principle, subcellular spatial resolution could be achieved with the VF-FLIM technique. 10.7554/eLife.44522.009Figure 1—source data 2.Properties of lifetime standards and VoltageFluor dyes.Fluorescein and erythrosin B standards were measured in drops of solution placed on a coverslip. For VF dyes, voltage sensitivities from intensity-based fluorescence imaging in HEK293T cells (%ΔF/F, percent change in fluorescence intensity for a voltage step from −60 mV to +40 mV) are from previously published work (Woodford et al., 2015). Lifetime data were obtained from voltage-clamp electrophysiology of HEK293T cells loaded with 100 nM VF. Lifetime listed here is the average 0 mV lifetime from the electrophysiology calibration. % Δτ/τ is the percent change in lifetime corresponding to a 100 mV step from −60 mV to +40 mV. Lifetime sample sizes: fluorescein 25, erythrosin B 25, VF2.1.Cl 17, VF2.0.Cl 17. For lifetime standards, each measurement was taken on a separate day. VF2.1.Cl data in HEK293T is duplicated in Figure 2—source data 1. Values are tabulated as mean ± SEM. 10.7554/eLife.44522.010Figure 1—source data 3.Comparison of optical approaches to absolute V_mem_ determination in HEK293T cells.Data are compiled from Figure 1 (VF-FLIM, this work), Figure 1—figure supplement 4 (CAESR; Brinks et al., 2015), and Figure 1—figure supplement 5 (Di-8-ANEPPS; Zhang et al., 1998). All data were obtained by simultaneous whole cell voltage clamp electrophysiology and optical recording in HEK293T (VF-FLIM n = 17 cells, CAESR n = 9, di-8-ANEPPS n = 16). Calculation of intra and inter cell accuracies are performed via root-mean-square deviation (RMSD) and discussed in detail in the Methods (see Resolution of VF-FLIM…). Regions of interest were chosen at the plasma membrane in all cases. Di-8-ANEPPS data are presented as the ratio of signal obtained with blue excitation to signal obtained with green excitation (R, see Materials and methods) and are not normalized to the 0 mV R.

Fluorescent voltage indicators that use photoinduced electron transfer (PeT) as a voltage-sensing mechanism are promising candidates for a FLIM-based approach to optical V_mem_ quantification. Because PeT affects the nonradiative decay rate of the fluorophore excited state, it has been successfully translated from intensity to τ_fl_ imaging with a number of small molecule probes for Ca^2+^ (Lakowicz et al., 1992). We previously established that VoltageFluor (VF)-type dyes transduce changes in cellular membrane potential to changes in fluorescence intensity and that the voltage response of VF dyes is consistent with a PeT-based response mechanism (Miller et al., 2012; Woodford et al., 2015). Changes in the transmembrane potential alter the rate of PeT (Li, 2007; de Silva et al., 1995) from an electron-rich aniline donor to a fluorescent reporter, thereby modulating the fluorescence intensity of VF dyes (Miller et al., 2012) (Figure 1A,B). VoltageFluors also display low toxicity and rapid, linear responses to voltage.

**Figure 1—figure supplement 1.**
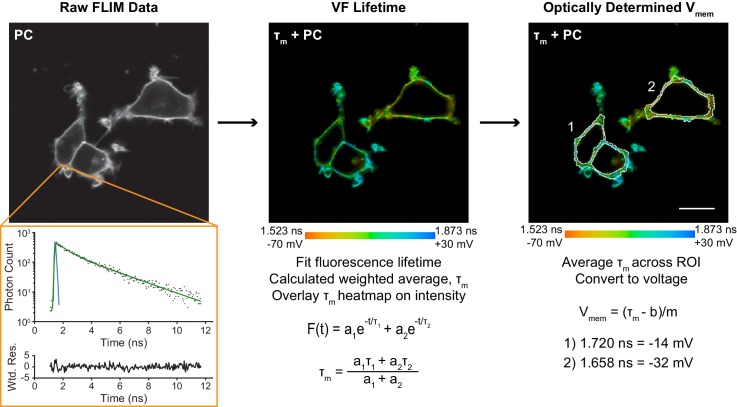
Overview of data processing to obtain membrane potential recordings from fluorescence lifetime. Time-correlated photon data (black dots, first panel) collected at each pixel were fit to an exponential decay model (green) with iterative reconvolution of the instrument response function (IRF, blue). The two components of the fluorescence lifetimes were converted to a weighted average (middle panel). Cell membranes (white outlines) were identified, and τ_m_ was averaged within each of these regions of interest (ROIs, right panel). These lifetimes were then converted to voltage via a previously determined lifetime-V_mem_ standard curve with slope m and y-intercept b. Additional details of this process are provided in the Methods. Wtd. Res.: weighted residuals of the fit, τ_m_: weighted average fluorescence lifetime, PC: photon count. τ_m_ + PC represents an overlay of the lifetime data (color heat map) onto the photon count image. Pixels that appear black in τ_m_ + PC images were below the required photon count threshold for fitting lifetime data; PC only images show photon counts without any such thresholds applied. Scale bar is 20 µm.

**Figure 1—figure supplement 2.**
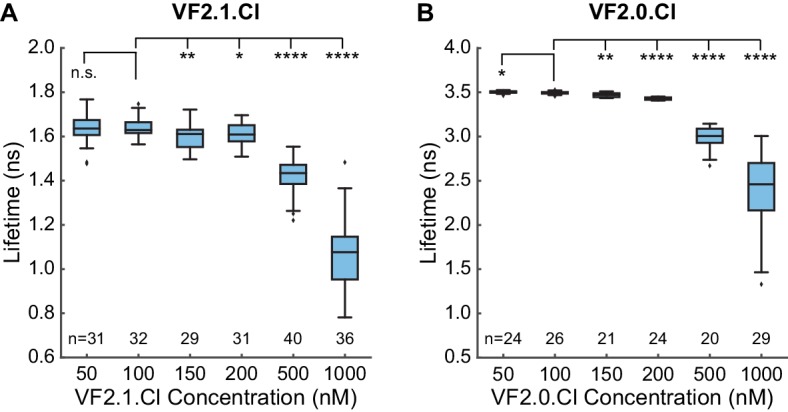
Concentration dependence of VoltageFluor lifetimes in HEK293T cells. Changes in lifetime arising from addition of a range of concentrations of (**A**) VF2.1.Cl or (**B**) voltage-insensitive control VF2.0.Cl in HEK293T cells. Biexponential fit models were used for all VF2.1.Cl concentrations and 1 μM VF2.0.Cl; a monoexponential model was used for all other VF2.0.Cl concentrations. Box plots represent the interquartile range, with whiskers and outliers determined with the Tukey method. Sample sizes indicate number of cell groups. Data were obtained over 2 to 4 different days from a total of 3 or 4 coverslips at each concentration. Asterisks indicate significant differences between the indicated concentration and the VF concentration used for electrophysiology experiments (n.s. p>0.05, *p<0.05, **p<0.01, ***p<0.001, ****p<0.0001, two-sided, unpaired, unequal variances t-test).

**Figure 1—figure supplement 3.**
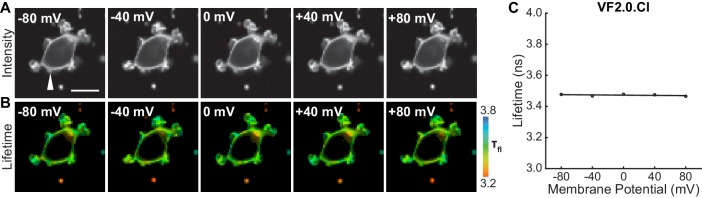
VF2.0.Cl lifetime does not depend on membrane potential. (**A**) Photon count and (**B**) lifetime images of a single HEK293T cell loaded with 100 nM VF2.0.Cl, with the membrane potential held at the indicated value via whole-cell voltage clamp electrophysiology. White arrow indicates patch pipette. Scale bar is 20 μm. (**C**) Quantification of images shown in (**B**) for this individual cell. Black line is the line of best fit.

**Figure 1—figure supplement 4.**
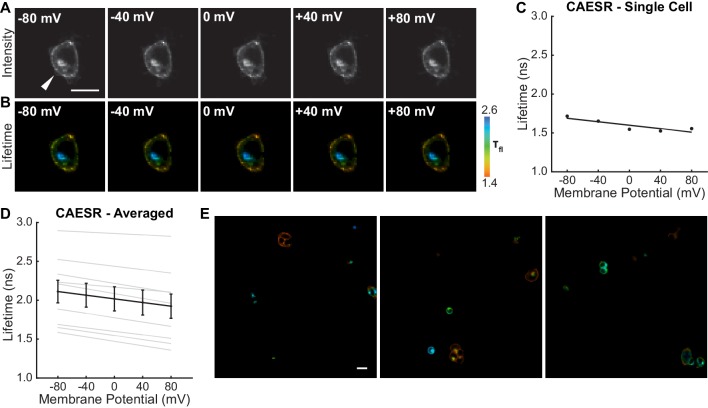
The GEVI CAESR shows variable lifetime-voltage relationships. (**A**) Photon count and (**B**) lifetime images of a HEK293T cell expressing CAESR and held at the indicated V_mem_ with voltage-clamp electrophysiology. White arrow indicates voltage-clamped cell. (**C**) Lifetime-V_mem_ relationship from the cell in (**B**), based on a single fit from combined fluorescence decays of all pixels in the cell membrane at each potential (see Materials and methods). Points indicate recordings at a given potential; solid line is line of best fit. (**D**) Evaluation of VF2.1.Cl lifetime-voltage relationships in many individual CAESR-expressing HEK293T cells. Gray lines represent linear fits on individual cells. Black line is the average fit across all cells (n = 9). (**E**) Representative lifetime images of CAESR in HEK293T cells. Scale bars represent 20 μm.

**Figure 1—figure supplement 5.**
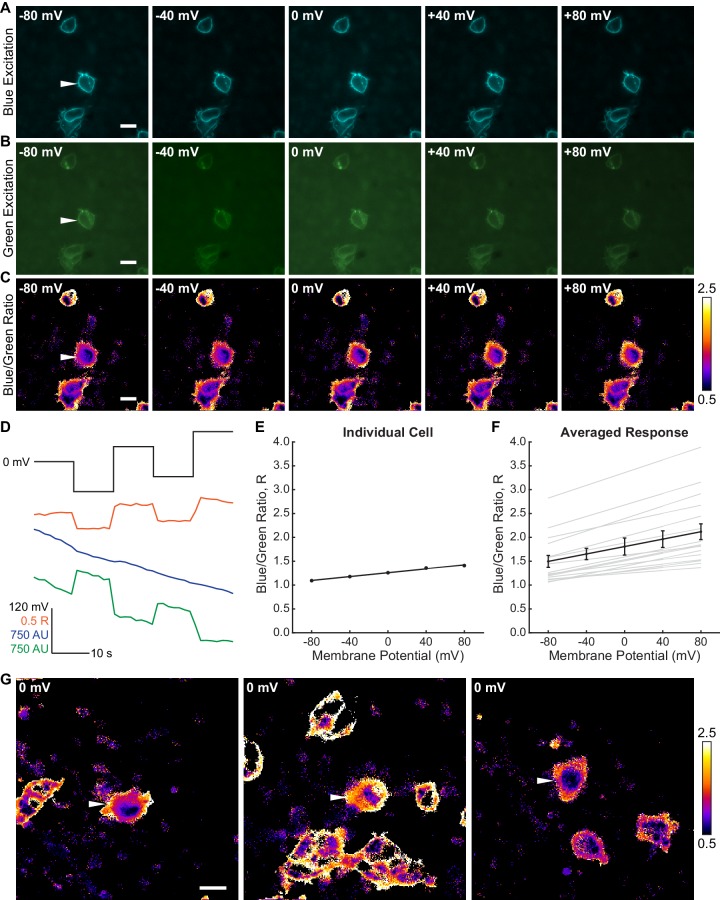
Ratiometric V_mem_ determinations with Di-8-ANEPPS in HEK293T cells. Different fluorescence excitation wavelengths (blue and green) were used to generate ratiometric images; emission wavelengths were constant throughout the experiment. Details of epifluorescence ratiometric imaging and data processing are described in the Materials and methods Simultaneous ratiometric imaging and whole cell patch clamp electrophysiology were used to determine the ratio between the blue and green excitation channels (blue/green ratio, R). Representative images of (**A**) blue-excited signal, (**B**) green-excited signal and (**C**) R of a HEK293T cell held at the indicated membrane potential with whole cell voltage clamp electrophysiology. White arrow indicates voltage clamped cell. Differences in background brightness between images are attributable to photobleaching of the probe; images were acquired in the order: 0,–80, +40,–40, +80 mV. (**D**) Time course of the voltage step protocol used for each cell (black line, top), along with a representative response in the fluorescence ratio (orange), blue excited fluorescence intensity (blue) and green excited fluorescence intensity (green). AU, arbitrary units. (**E**) R at various potentials for an individual HEK293T cell (black dots), along with an R-V_mem_ line of best fit (black line). Values shown are the average of all ratio images from a particular cell at a given potential, excluding the first and last ratio image taken for each V_mem_ value. Data shown in (**D**) and (**E**) are from the cell depicted in (**A**)-(**C**). (**F**) Aggregated lines of best fit (gray) for R-V_mem_ calibrations on multiple HEK293T cells. Average response is shown in black; ratios at each potential are depicted as mean ± SEM (n = 16 cells). (**G**) Additional representative R images of Di-8-ANEPPS in HEK293T cells. The cell indicated with the white arrow is held at 0 mV with electrophysiology; other cells are unperturbed. Images represent one frame at the given voltage and are not averaged. Regions of interest (ROIs) were drawn around the membrane only, avoiding both the internalized dye signal and the artificially high ratios just outside the cells (which appear as white edges) as much as possible. Scale bars represent 20 µm.

Here, we develop fluorescence lifetime imaging of VoltageFluor dyes (VF-FLIM) as a quantitative, all-optical approach for recording absolute membrane potential with single cell resolution. Using patch-clamp electrophysiology as a standard, we demonstrate that VF-FLIM reports absolute membrane potential in single trials with 10 to 23 mV accuracy (root mean square deviation, RMSD; 15 s acquisition), depending on the cell line. In all cases tested, VF-FLIM tracks membrane potential *changes* with better than 5 mV accuracy (RMSD). We benchmark VF-FLIM against previously reported optical absolute V_mem_ recording approaches and demonstrate resolution improvements of 8-fold over ratiometric strategies and 19-fold over other lifetime-based strategies. To highlight the increased throughput relative to manual patch-clamp electrophysiology, we document resting membrane potentials of thousands of cells. To our knowledge, this work represents the first broad view of the distribution of resting membrane potentials present in situ. VF-FLIM is limited to acquisition speeds on the order of seconds, but it is well-suited for studying gradual V_mem_ dynamics. Using VF-FLIM, we quantify and track the evolution of a 10–15 mV V_mem_ hyperpolarization over minutes following epidermal growth factor (EGF) stimulation of human carcinoma cells. Through pharmacological perturbations, we conclude that the voltage changes following EGF stimulation arise from activation of the calcium-activated potassium channel K_Ca_3.1. Our results show that fluorescence lifetime of VF dyes is a generalizable and effective approach for studying resting membrane potential in a range of cell lines (Lakowicz et al., 1992).

## Results

### VoltageFluor fluorescence lifetime varies linearly with membrane potential

**Scheme 1.**
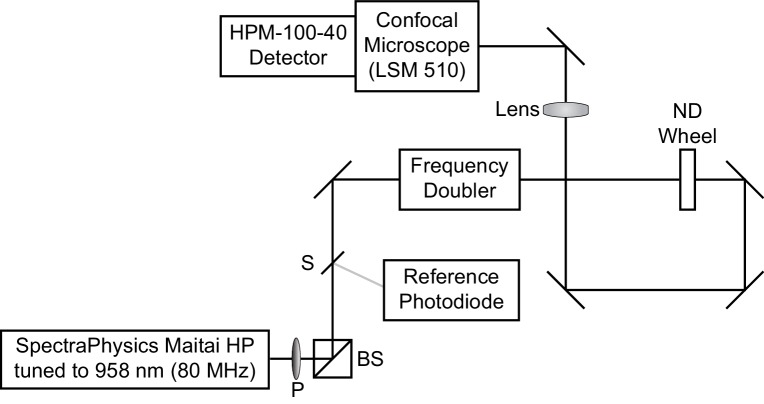
Optical diagram for time correlated single photon counting microscope. Excitation light was supplied by a Ti:Sapphire laser tuned to 958 nm. A small amount of light was redirected by a beam sampler (S) to a reference photodiode. The remaining light was passed through a frequency doubler to obtain 479 nm excitation light, which entered the LSM510 confocal microscope. A polarizer (P) followed by a polarizing beamsplitter (BS), as well as a neutral density (ND) wheel, allowed control of the amount of light passed to the sample.

To characterize how the photoinduced electron transfer process affects fluorescence lifetime, we compared the τ_fl_ of the voltage-sensitive dye VF2.1.Cl with its voltage-insensitive counterpart VF2.0.Cl (Figure 1B). We recorded the τ_fl_ of bath-applied VF dyes in HEK293T cells using time-correlated single-photon counting (TCSPC) FLIM (Figure 1C–E, Scheme 1). VF2.1.Cl is localized to the plasma membrane and exhibits a biexponential τ_fl_ decay with decay constants of approximately 0.9 and 2.6 ns (Figure 1—figure supplement 1). For all subsequent analysis of VF2.1.Cl lifetime, we refer to the weighted average τ_fl_, which is approximately 1.6 ns in HEK293T cell membranes at rest. VF2.0.Cl (Figure 1B), which lacks the aniline substitution and is therefore voltage-insensitive (Woodford et al., 2015), shows a τ_fl_ of 3.5 ns in cell membranes, which is similar to the lifetime of an unsubstituted fluorescein (Magde et al., 1999) (Figure 1—source data 2). We also examined VoltageFluor lifetimes at a variety of dye loading concentrations to test for concentration-dependent changes in dye lifetime, which have been reported for fluorescein derivatives (Chen and Knutson, 1988). Shortened VF lifetimes were observed at high dye concentrations (Figure 1—figure supplement 2); all subsequent VF-FLIM studies were conducted at dye concentrations low enough to avoid this concentration-dependent change in lifetime.

**Scheme 2.**
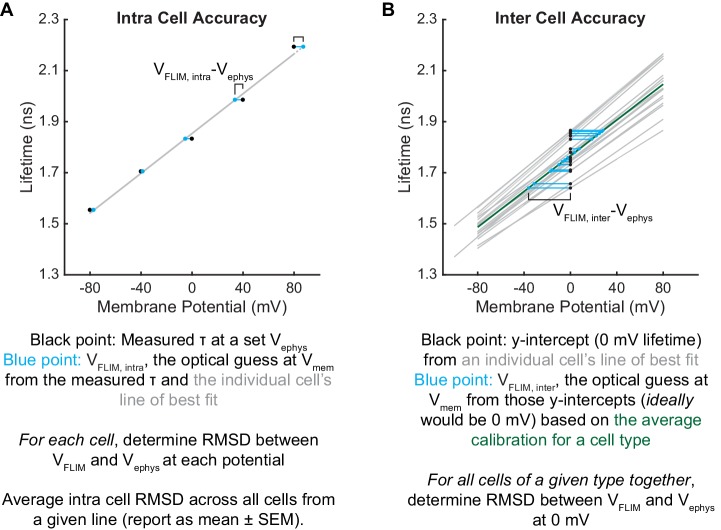
Intra and inter-cell V_mem_ resolution calculations. Data are taken directly from Figure 1H,I as an example. (**A**) Intra cell values are the RMSD between the voltage equivalent of the measured lifetime (V_FLIM_) and voltage set by electrophysiology (V_ephys_). V_FLIM_ values are calculated using that particular cell’s line of best fit, so one value is obtained per cell. Here, we present intra cell error as the mean ± SEM of all cells from a given cell line. (**B**) Inter cell errors are the RMSD between the voltage-equivalent of the 0 mV lifetime for all cells tested from a cell line (V_FLIM_, determined with the average slope and y-intercept for that cell line) and the ground truth value of 0 mV. Inter-cell accuracy is calculated from all of the calibration data for a cell line, so there is one value per cell line. Black points are experimental y-intercepts and blue points are the V_FLIM_ optical voltage determinations from those lifetimes. Gray lines are lines of best fit for individual cells. Green line in (B) represents the average τ_fl_-V_mem_ relationship for a cell line.

To assess the voltage dependence of VoltageFluor τ_fl_, we controlled the plasma membrane potential of HEK293T cells with whole-cell voltage-clamp electrophysiology while simultaneously measuring the τ_fl_ of VF2.1.Cl (Figure 1C). Single-cell recordings show a linear τ_fl_ response to applied voltage steps, and individual measurements deviate minimally from the linear fit (Figure 1F–H). VF2.1.Cl τ_fl_ is reproducible across different cells at the same resting membrane potential, allowing determination of V_mem_ from τ_fl_ images taken without concurrent electrophysiology (Figure 1I). Voltage-insensitive VF2.0.Cl shows no τ_fl_ change in response to voltage (Figure 1J, Figure 1—figure supplement 3), consistent with a τ_fl_ change in VF2.1.Cl arising from a voltage-dependent PeT process. In HEK293T cells, VF2.1.Cl exhibits a sensitivity of 3.50 ± 0.08 ps/mV and a 0 mV lifetime of 1.77 ± 0.02 ns, corresponding to a fractional change in τ_fl_ (Δτ/τ) of 22.4 ± 0.4% per 100 mV. These values are in good agreement with the 27% ΔF/F intensity change per 100 mV originally observed for VF2.1.Cl (Miller et al., 2012; Woodford et al., 2015). Because %ΔF/F is a fluorescence intensity-based metric, it cannot be used to measure absolute V_mem_; however, agreement between %ΔF/F and %Δτ/τ is consistent with a PeT-based V_mem_ sensing mechanism in VFs. To estimate the voltage resolution of VF-FLIM, we analyzed the variability in successive measurements on the same cell (intra-cell resolution) and on different cells (inter-cell resolution, see Materials and methods). We estimate that the resolution for tracking and quantifying voltage changes in a single HEK293T cell is 3.5 ± 0.4 mV (intra-cell resolution, average RMSD from each electrophysiological calibration, Scheme 2), whereas the resolution for single-trial determination of a particular HEK293T cell’s absolute V_mem_ is 19 mV (inter-cell resolution, RMSD of each calibration slope to the average calibration, Scheme 2) within a 15 s bandwidth.

We compared the performance of VF-FLIM in HEK293T cells to that of two previously documented strategies for optical absolute V_mem_ determination. We first tested the voltage resolution of CAESR, the best previously reported GEVI for recording absolute V_mem_ with FLIM (Brinks et al., 2015). Using simultaneous FLIM and voltage-clamp electrophysiology, we determined the relationship between τ_fl_ and V_mem_ for CAESR under one photon excitation (Figure 1—figure supplement 4). We recorded a sensitivity of −1.2 ± 0.1 ps/mV and a 0 mV lifetime of 2.0 ± 0.2 ns, which corresponds to a −6.1 ± 0.8% Δτ/τ per 100 mV (mean ± SEM of 9 measurements), in agreement with the reported sensitivity of −0.9 ps/mV and 0 mV lifetime of 2.7 ns with 2 photon excitation (Brinks et al., 2015). Relative to VF2.1.Cl, CAESR displays 3-fold lower sensitivity (−1.2 ps/mV vs 3.5 ps/mV in HEK293T cells) and 7-fold higher voltage-independent variability in lifetime (0.46 ns vs 0.07 ns, standard deviation of the 0 mV lifetime measurement). For CAESR in HEK293T cells, we calculate a voltage resolution of 33 ± 7 mV for quantifying voltage changes on an individual cell (intra-cell RMSD, compared to 3.5 mV for VF2.1.Cl, see Materials and methods) and resolution of 370 mV for determination of a particular cell’s absolute V_mem_ (inter-cell RMSD, compared to 19 mV for VF2.1.Cl).

We also measured the absolute voltage resolution of the ratio-based sensor di-8-ANEPPS, which reports membrane potential by the wavelength of its excitation and emission spectra (Loew et al., 1979). Ratio-based imaging can be achieved by comparing the fluorescence emission at different excitation wavelengths (Zhang et al., 1998); here, we used the ratio, R, of the blue-excited emission to the green-excited emission (see Materials and methods). Via simultaneous ratio imaging and whole cell voltage clamp electrophysiology, we record a sensitivity of 0.0039 ± 0.0004 R per mV, with a y-intercept (0 mV) R value of 1.8 ± 0.2 (Figure 1—figure supplement 5; mean ± SEM of n = 16 HEK293T cells). R depends on the excitation and emission conditions used but should be relatively reproducible on a given microscope rig. To compare R from our system with previous work, we normalized all R values to the R value at 0 mV for each cell. Using the above data, we obtain a sensitivity of 0.0022 ± 0.0002 normalized R per mV, with a 0 mV normalized R of 1.02 ± 0.02, in good agreement with reported values (0.0015 normalized R per mV) (Zhang et al., 1998). For analysis of voltage resolution, we compare VF-FLIM to the non-normalized R, since normalization requires an electrode-based measurement for every recording and is thus not a truly optical strategy. From the non-normalized di-8-ANEPPS R, we obtain an intra-cell resolution (RMSD) of 18 ± 3 mV (5-fold less accurate than VF-FLIM) and an inter-cell resolution (RMSD) of 150 mV (8-fold less accurate than VF-FLIM). The sensitivities and resolutions of VF-FLIM, CAESR, and di-8-ANEPPS in HEK293T are tabulated in Figure 1—source data 3. Because cellular resting membrane potentials and voltage changes (e.g. action potentials) are on the order of tens of millivolts, the resolution improvements achieved by VF-FLIM enable biologically relevant absolute V_mem_ recordings: impossible with previous approaches.

### Evaluation of VF-FLIM across cell lines and culture conditions

**Figure 2.**
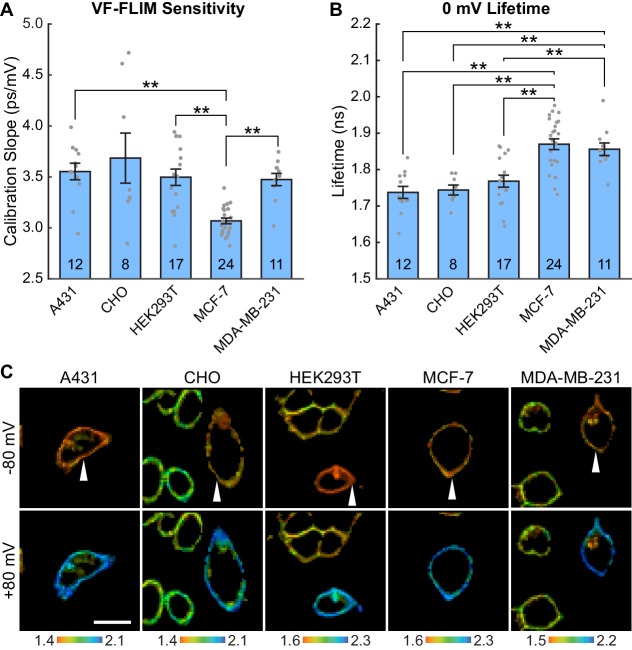
VF-FLIM is a general and portable method for optically determining membrane potential. VF2.1.Cl lifetime-voltage relationships were determined with whole cell voltage clamp electrophysiology in five cell lines. (**A**) Slopes of the linear fits for single cell lifetime-voltage relationships, shown as mean ± S.E.M. Gray dots indicate results from individual cells. Statistically significant differences exist between groups (One-way ANOVA with Welch’s correction: F(4, 23.07)=18.12, p<0.0001). Data were tested for normality (Shapiro-Wilk test, p>0.05 for all cell lines) and homoscedasticity (Levene’s test on the median, F(4,67) = 5.07, p=0.0013). ** indicates p<0.01; if significance is not indicated, p>0.05 (Games-Howell post hoc test). (**B**) 0 mV reference point of linear fits for the lifetime-voltage relationship, shown as mean ± S.E.M. Gray dots indicate results from individual cells. Significant differences exist between groups (One-way ANOVA: F(4, 67)=14.43, p<0.0001). Data were tested for normality (Shapiro-Wilk test, p>0.05 for all cell lines) and homoscedasticity (Levene’s test on the median, F(4,67) = 1.29, p=0.28). ** indicates p<0.01; if significance is not indicated, p>0.05 (Tukey-Kramer post hoc test). (**C**) Representative lifetime-intensity overlay images for each cell line with the indicated cells (white arrow) held at −80 mV (top) or +80 mV (bottom). Lifetime scales are in ns. Scale bar is 20 μm. 10.7554/eLife.44522.018Figure 2—source data 1.Lifetime-V_mem_ standard curves for VF2.1.Cl lifetime in various cell lines.Whole-cell voltage-clamp electrophysiology was used to determine the relationship between VF2.1.Cl lifetime and membrane potential in five different cell lines. Parameters of this linear model are listed above. The %Δτ/τ is the percent change in the lifetime observed for a voltage step from −60 mV to +40 mV. The intra-cell RMSD represents the accuracy for quantifying voltage changes in a particular cell (see Materials and methods). The inter-cell RMSD represents the expected variability in single-trial absolute V_mem_ determinations. Sample sizes: A431 12, CHO 8, HEK293T 17, MCF-7 24, MDA-MB-231 11. All values are tabulated as mean ± SEM.

To test the generalizability of VF-FLIM, we determined τ_fl_-V_mem_ calibrations in four additional commonly used cell lines: A431, CHO, MDA-MB-231, and MCF-7 (Figure 2, Figure 2—figure supplement 1, Figure 2—figure supplement 2). We observe a linear τ_fl_ response in all cell lines tested. The slope (voltage sensitivity) and y-intercept (0 mV lifetime) of the τ_fl_-V_mem_ response varied slightly across cell lines, with average sensitivities of 3.1 to 3.7 ps/mV and average 0 mV lifetimes ranging from 1.74 to 1.87 ns. In all cell lines, we observed better voltage resolution for quantification of V_mem_ changes on a given cell versus comparisons of absolute V_mem_ between cells. Changes in voltage for a given cell could be quantified with resolutions at or better than 5 mV (intra-cell resolution, Materials and methods). For absolute V_mem_ determination of a single cell, we observed voltage resolutions ranging from 10 to 23 mV (inter-cell resolution, 15 s acquisition time, Figure 2—source data 1). Statistically significant differences among the cell lines tested were observed for cellular τ_fl_-V_mem_ calibrations in both the slope (One-way ANOVA with Welch’s correction: F(4, 23.07)=18.12, p<0.0001) and average 0 mV lifetime (One-way ANOVA: F(4, 67)=14.43, p<0.0001). There were no statistically significant differences between A431, CHO, and HEK293T cells (p>0.05, Games-Howell and Tukey-Kramer post hoc tests for the slope and 0 mV lifetime respectively). MDA-MB-231 and MCF-7 cells showed statistically significant variability from other cell lines in slope and/or 0 mV lifetime.

**Figure 2—figure supplement 1.**
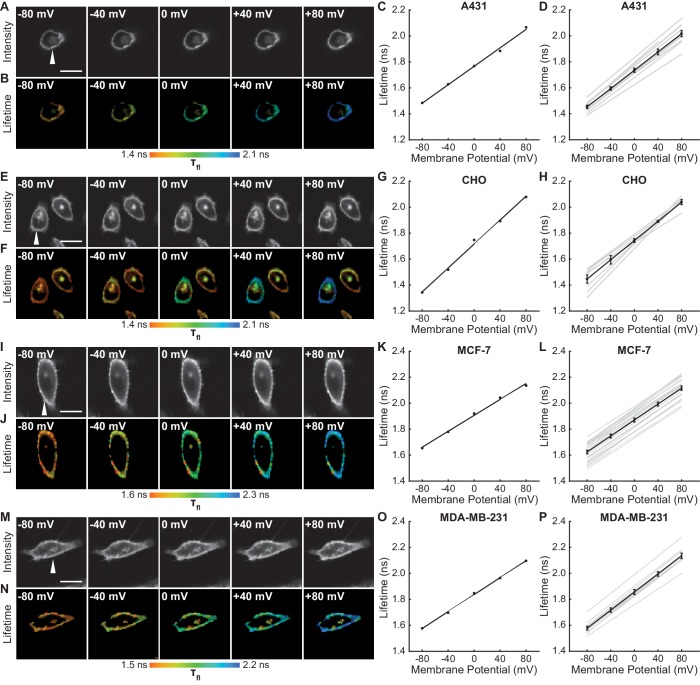
VoltageFluor lifetime reports voltage in diverse cell lines. (**A**) Representative photon count and (**B**) lifetime images of a VF2.1.Cl in A431 cells with V_mem_ held at the indicated value with voltage-clamp electrophysiology. A431 cells were not serum starved for these experiments. (**C**) Quantification of the images in (**B**), with the line of best fit for this single trial. (**D**) Lines of best fit for the lifetime-V_mem_ relationships of 12 A431 cells (gray lines). Average lifetime at each potential is shown as mean ± SEM, with the average line of best fit in black. (**E**)-(**H**) Lifetime-V_mem_ standard curve determination in CHO cells (n = 8). (**I**)-(**L**) Lifetime-V_mem_ standard curve determination in MCF-7 cells (n = 24). (**M**)-(**P**) Lifetime-V_mem_ standard curve determination in MDA-MB-231 cells (n = 11). VF2.1.Cl concentration was 100 nM in all cases. White arrows indicates the voltage-clamped cell. Scale bars are 20 μm..

**Figure 2—figure supplement 2.**
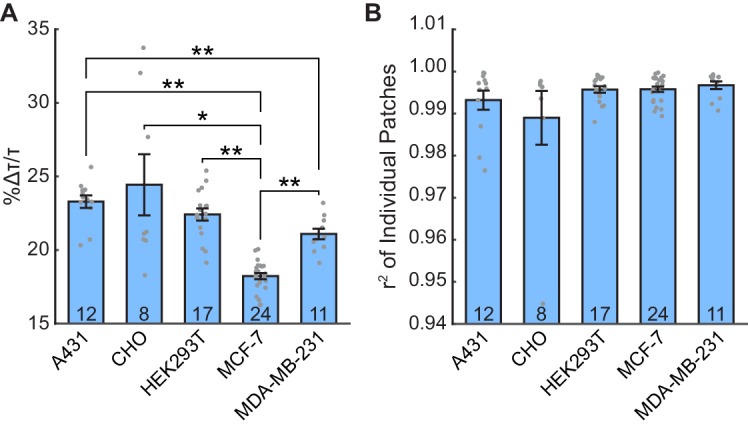
Additional parameters of linear lifetime-voltage standard curves. (**A**) Percent change in VF2.1.Cl lifetime per 100 mV change in voltage, relative to the lifetime at −60 mV. Significant differences exist between cell lines (one-way ANOVA with Welch’s correction, F(4,24.08) = 41.75, p<0.0001). Data were tested for normality (Shapiro-Wilk test, p>0.05 for all cell lines) and homoscedasticity (Levene’s test on the median, F(4,67) = 5.74, p=0.00049). Asterisks indicate statistically significant differences (*p<0.05, **p<0.01, Games-Howell post hoc test). (**B**) Correlation coefficients (r^2^) for the lines of best fit of VF2.1.Cl lifetime versus membrane potential. No significant differences exist in r^2^values between cell lines (Kruskal-Wallis test, H = 3.20, 4 degrees of freedom, p=0.53). Data were tested for normality (Shapiro-Wilk test, p<0.05 for 4 of 5 cell lines) and homoscedasticity (Levene’s test on the median, F(4,67) = 1.55, p=0.20). In both (**A**) and (**B**), data are shown as mean ± S.E.M., with gray dots indicating values from individual patches.

**Figure 2—figure supplement 3.**
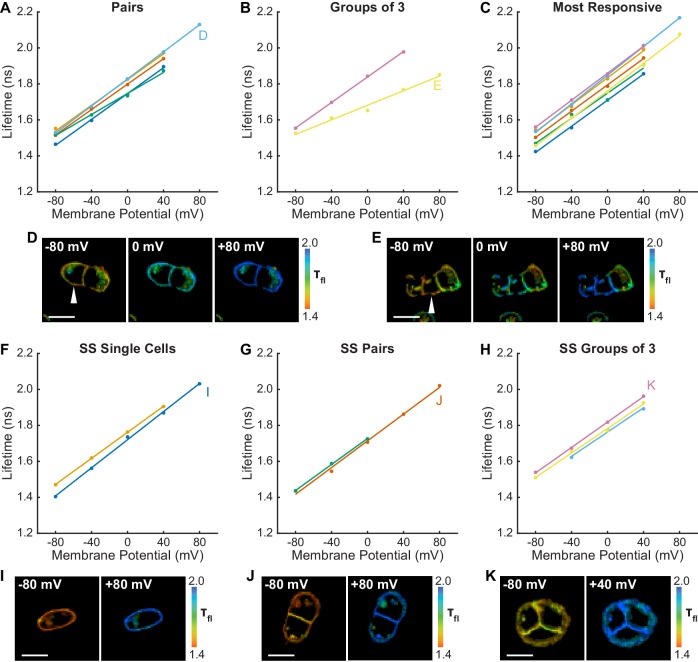
Relationship between lifetime and membrane potential extends to groups of cells and across culture conditions. Electrophysiological calibration of lifetime was performed on small groups of A431 cells and on serum starved (SS) A431 cells to verify that the V_mem_-lifetime standard curves for a given cell line are generalizable across many cellular growth conditions. For all graphs, each line represents a group of cells. Letters on the graphs indicate the subfigure where images from that recording are shown. (**A**) Lifetime-voltage relationships in cell pairs, in which only one cell was directly controlled with voltage-clamp electrophysiology. (**B**) Lifetime-voltage relationships in groups of three cells, in which only one cell was directly controlled with voltage-clamp electrophysiology. (**C**) Lifetime for the most responsive cell from pairs and groups of three in (**A**) and (**B**). Line color codes are maintained from (**A**) and (**B**). (**D, E**) Representative lifetime images from (**A**) and (**B**) respectively. White arrow indicates cell directly controlled with electrophysiology. (**F**) Lifetime-voltage relationship in SS single cells, (**G**) pairs, and (**H**) groups of three cells. (**I**)-(**K**) Representative images from (**F**)-(**H**). Scale bars are 20 μm.

**Figure 2—figure supplement 4.**
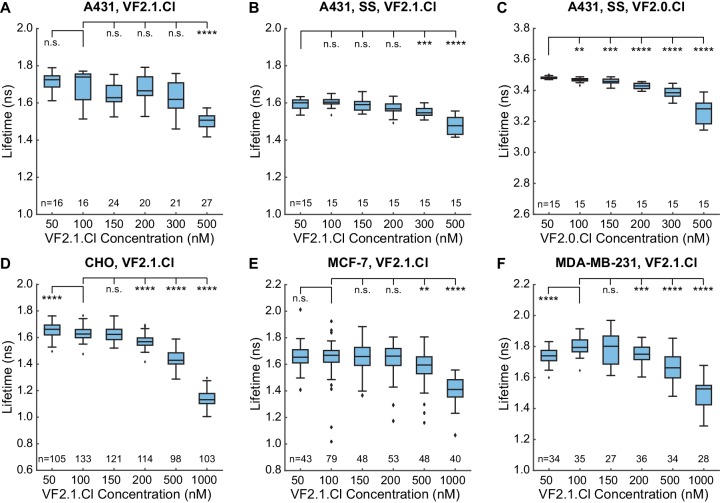
Concentration dependence of VoltageFluor lifetime in four cell lines. A431 cells were analyzed with VF2.1.Cl both in (**A**) full serum and (**B**) serum-starved conditions. (**C**) VF2.0.Cl in serum-starved A431 cells. (**D**) VF2.1.Cl in CHO cells. (**E**) VF2.1.Cl in MCF-7 cells. (**F**) VF2.1.Cl in MDA-MB-231 cells. All VF2.1.Cl data were fit with a biexponential model, and all VF2.0.Cl data were fit with a monoexponential model. Box plots represent the interquartile range, with whiskers and outliers determined with the Tukey method. Sample sizes indicate number of cell groups. Data were acquired over 2 to 4 different days from a total of 3 or 4 coverslips at each concentration. Asterisks indicate significant differences between the indicated concentration and the VF concentration selected for additional experiments (n.s. p>0.05, *p<0.05, **p<0.01, ***p<0.001, ****p<0.0001, two-sided, unpaired, unequal variances t-test).

To verify that VF-FLIM was robust in groups of cells in addition to the isolated, single cells generally used for patch clamp electrophysiology, we determined lifetime-voltage relationships for small groups of A431 cells (Figure 2—figure supplement 3A–E). We found that calibrations made in small groups of cells are nearly identical to those obtained on individual cells, indicating that VF-FLIM only needs to be calibrated once for a given type of cell. For pairs or groups of three cells we recorded a sensitivity of 3.3 ± 0.2 ps/mV and a 0 mV lifetime of 1.78 ± 0.02 ns (mean ± SEM of 7 cells (5 pairs and 2 groups of 3); values are for the entire group, not just the cell in contact with the electrode), which is similar to the sensitivity of 3.55 ± 0.08 ps/mV and 0 mV lifetime of 1.74 ± 0.02 ns we observe in single A431 cells. The slight reduction in sensitivity seen in cell groups is likely attributable to space clamp error, which prevents complete voltage clamp of the cell group (Williams and Mitchell, 2008; Armstrong and Gilly, 1992). Indeed, when we analyzed only the most responsive cell in the group (in contact with the electrode), we obtained a slope of 3.7 ± 0.1 ps/mV and 0 mV lifetime of 1.79 ± 0.02 ns, in good agreement with the single cell data. The space clamp error can be clearly visualized in Figure 2—figure supplement 3E, where one cell in the group of 3 responded much less to the voltage command.

To test whether VF-FLIM is also extensible to cells maintained with different culture conditions, we recorded lifetime-V_mem_ relationship in serum-starved A431 cells (Figure 2—figure supplement 3F–K), obtaining an average sensitivity of 3.6 ± 0.1 ps/mV and a 0 mV lifetime of 1.76 ± 0.01 ns (n = 7; two single cells, two pairs, 3 groups of 3 cells; values are average lifetime across the whole cell group), in excellent agreement with the values obtained for non-serum starved cells. We also tested for concentration-dependent changes in VF lifetime in all five cell lines and in serum starvation conditions. Similar to VF2.1.Cl lifetime in HEK293T cells (Figure 1—figure supplement 2), we observed shortening of VF2.1.Cl lifetimes beginning between 200 and 500 nM dye in all cases (Figure 2—figure supplement 4). All subsequent experiments were carried out at VF2.1.Cl concentrations well below the regime where VF concentration-dependent lifetime changes were observed.

### Optical determination of resting membrane potential distributions

**Figure 3.**
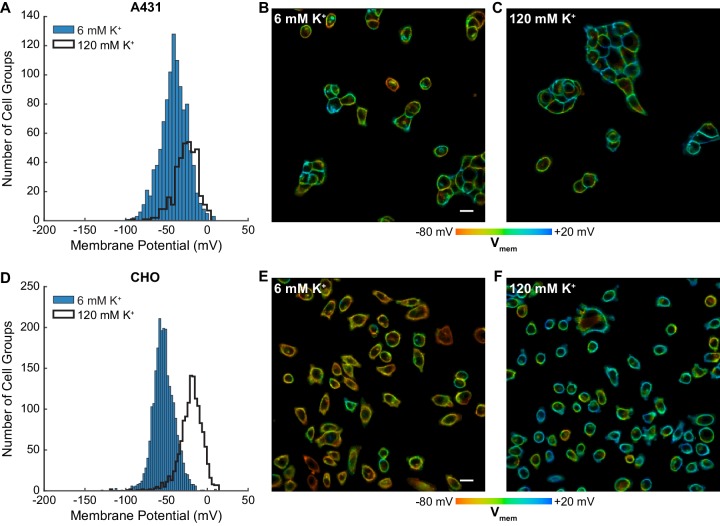
Rapid optical profiling of V_mem_ at rest and in high extracellular K^+^. Fluorescence lifetime images of cells incubated with 100 nM VF2.1.Cl were used to determine V_mem_ from previously performed electrophysiological calibration (Figure 2). (**A**) Histograms of V_mem_ values recorded in A431 cells incubated with 6 mM extracellular K^+^ (commercial HBSS, n = 1056) or 120 mM K^+^ (high K^+^ HBSS, n = 368). (**B**) Representative lifetime image of A431 cells in 6 mM extracellular K^+^. (**C**) Representative lifetime image of A431 cells in 120 mM extracellular K^+^. (**D**) Histograms of V_mem_ values observed in CHO cells under normal (n = 2410) and high K^+^ (n = 1310) conditions. Representative lifetime image of CHO cells in (**E**) 6 mM and (**F**) 120 mM extracellular K^+^. Histogram bin sizes were determined by the Freedman-Diaconis rule. Intensities in the lifetime-intensity overlay images are not scaled to each other. Scale bars, 20 μm. 10.7554/eLife.44522.022Figure 3—source data 1.V_mem_ measurements made with VF-FLIM agree with previously reported values.Comparison of optically-determined resting membrane potential values (in millivolts) and previously reported values. This table summarizes data presented in Figure 3 and Figure 3—figure supplement 1. Optically determined membrane potentials were calculated from lifetime-V_mem_ standard curves (Figure 2—source data 1). For tabulated literature values, measures of error and central tendency were used from the original publication. In some cases, none were given or only ranges were discussed. The mean of the reported ephys values is the mean of the values listed here. Sample sizes for resting and elevated K^+^, respectively: A431 1056, 368; CHO 2410, 1310; HEK293T 1613, 520; MCF-7 1259, 681; MDA-MB-231 1840, 558.

The throughput of VF-FLIM enables cataloging of resting membrane potentials of thousands of cells in only a few hours of the experimenter’s time. We optically recorded resting membrane potential distributions for A431, CHO, HEK293T, MCF-7, and MDA-MB-231 cells using VF-FLIM (Figure 3, Figure 3—figure supplement 1, Figure 3—figure supplement 2). We report resting membrane potentials by cell group (Materials and methods, Figure 1—figure supplement 1) because adjacent cells in these cultures are electrically coupled to some degree via gap junctions (Meşe et al., 2007). Each group of cells represents an independent sample for V_mem_. In addition, the fluorescent signal originating from membranes of adjacent cells cannot be separated with a conventional optical microscope, so assignment of a region of membrane connecting multiple cells would be arbitrary. VF-FLIM images (Figure 3, Figure 3—figure supplement 1, Figure 3—figure supplement 2) contain spatially resolved voltage information, but caution should be employed in interpreting pixel to pixel differences in lifetime. Because VF-FLIM was calibrated here using the average plasma membrane τ_fl_ for each cell, optical V_mem_ should be interpreted per cell or cell group.

**Figure 3—figure supplement 1.**
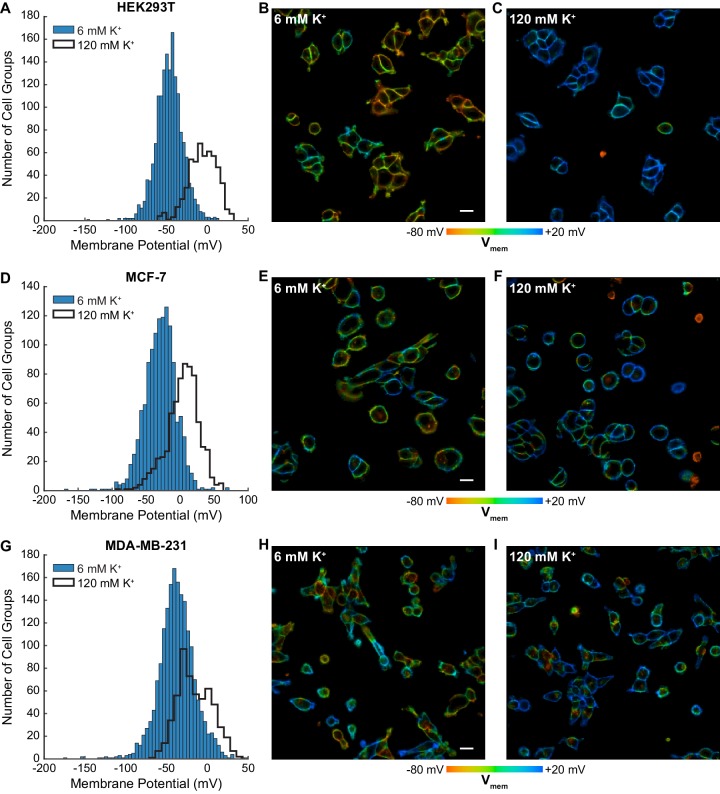
Optically recorded V_mem_ distributions in HEK293T, MCF-7 and MDA-MB-231 cells. Fluorescence lifetime images of cells incubated with 100 nM VF2.1.Cl were used to determine V_mem_ from previously performed electrophysiological calibration (Figure 2). (**A**) Histograms of V_mem_ values recorded in HEK293T cells incubated with 6 mM extracellular K^+^ (commercial HBSS, n = 1613) or 120 mM K^+^ (high K^+^ HBSS, n = 520). (**B**) Representative lifetime image of HEK293T cells with 6 mM extracellular K^+^. (**C**) Representative lifetime image of HEK293T cells in 120 mM extracellular K^+^. (**D**) Histograms of V_mem_ values observed in MCF-7 cells under normal (n = 1259) and high K^+^ (n = 681) conditions. Representative lifetime images of MCF-7 cells in (**E**) 6 mM and (**F**) 120 mM extracellular K^+^. (**G**) Histograms of V_mem_ values observed in MDA-MB-231 cells under normal (n = 1840) and high K^+^ (n = 558) conditions. Representative lifetime images of MDA-MB-231 cells in (**H**) 6 mM and (**I**) 120 mM extracellular K^+^. Histogram bin sizes were determined by the Freedman-Diaconis rule. Intensities in the lifetime-intensity overlay images are not scaled to each other. Scale bars, 20 μm.

**Figure 3—figure supplement 2.**
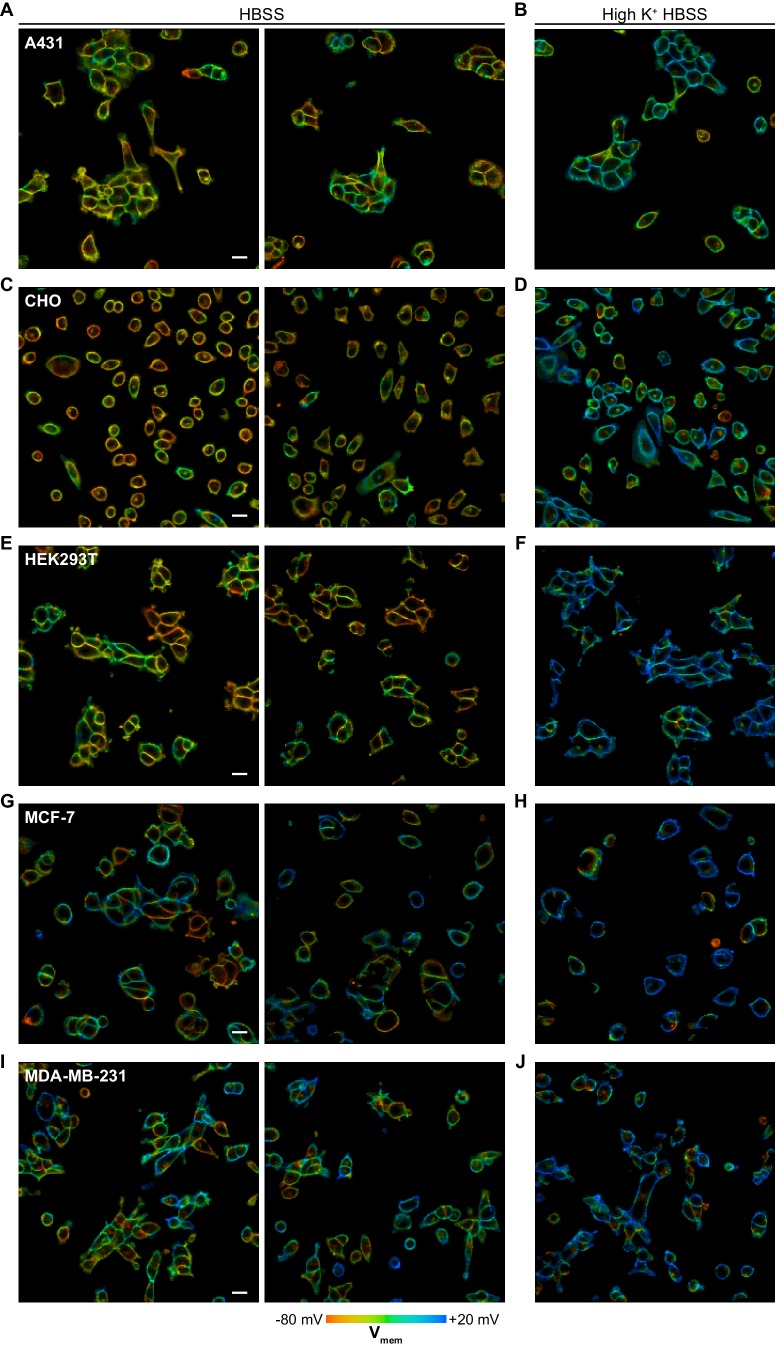
Representative images of cultured cell resting membrane potential. Representative VF-FLIM images of cells in standard imaging buffer (HBSS, 6 mM extracellular K^+^) and high K^+^ imaging buffer (high K^+^ HBSS, 120 mM extracellular K^+^). Membrane potential was calculated per cell group; analyses of pixel by pixel differences in lifetime fall beyond the resolution limit of the VF-FLIM calibrations in this work. Images depict A431 cells in (**A**) HBSS and (**B**) high K^+^ HBSS, CHO cells in (**C**) HBSS and (**D**) high K^+^ HBSS, HEK293T cells in (**E**) HBSS and (**F**) high K^+^ HBSS, MCF-7 cells in (**G**) HBSS and (**H**) high K^+^ HBSS, and MDA-MB-231 cells in (**I**) HBSS and (**J**) high K^+^ HBSS.

Mean resting membrane potentials recorded by VF-FLIM range from −53 to −29 mV, depending on the cell line. These average V_mem_ values fall within the range reported in the literature for all of the cell lines we measured (Figure 3—source data 1). We also recorded resting membrane potentials in a high K^+^ buffer (120 mM K^+^, ‘high K^+^ HBSS’), where we observed a depolarization of 15 to 41 mV, bringing the mean V_mem_ up to −26 mV to +4 mV, again depending on the cell line. Although 120 mM extracellular K^+^ should be strongly depolarizing, it will not necessarily produce a membrane potential of 0 mV. Because few literature reports of electrophysiological measurements in 120 mM K^+^ exist as a point of comparison, we obtained a rough estimate of V_mem_ in 6 mM extracellular K^+^ and 120 mM extracellular K^+^ using the Goldman-Hodgkin-Katz (GHK) equation (Hodgkin and Katz, 1949). Under our imaging conditions and with a broad range of possible ion permeabilities and intracellular ion concentrations, the GHK equation allows V_mem_ ranging from −91 to −27 mV in 6 mM extracellular K^+^ and −25 to +2 mV in 120 mM extracellular K^+^ (see Materials and methods). Recorded VF-FLIM values fall well within this allowed range. Notably, although the GHK equation can determine ranges of reasonable V_mem_ values, GHK-based V_mem_ results are approximate at best because of the difficulty in obtaining accurate values of permeabilities and intracellular ion concentrations for specific cell lines. Direct measurement of V_mem_, rather than theoretical calculation, is required to obtain accurate values.

### Membrane potential dynamics in epidermal growth factor signaling

We thought VF-FLIM was a promising method for elucidating the roles of membrane potential in non-excitable cell signaling. Specifically, we wondered whether VF-FLIM might be well-suited to dissect conflicting reports surrounding changes in membrane potential during EGF/EGF receptor (EGFR)-mediated signaling. Receptor tyrosine kinase (RTK)-mediated signaling is a canonical signaling paradigm for eukaryotic cells, transducing extracellular signals into changes in cellular state. Although the involvement of second messengers like Ca^2+^, cyclic nucleotides, and lipids are well characterized, membrane potential dynamics and their associated roles in non-excitable cell signaling remain less well-defined. In particular, the activation of EGFR via EGF has variously been reported to be depolarizing (Rothenberg et al., 1982), hyperpolarizing (Pandiella et al., 1989), or electrically silent (Moolenaar et al., 1982; Moolenaar et al., 1986).

**Figure 4.**
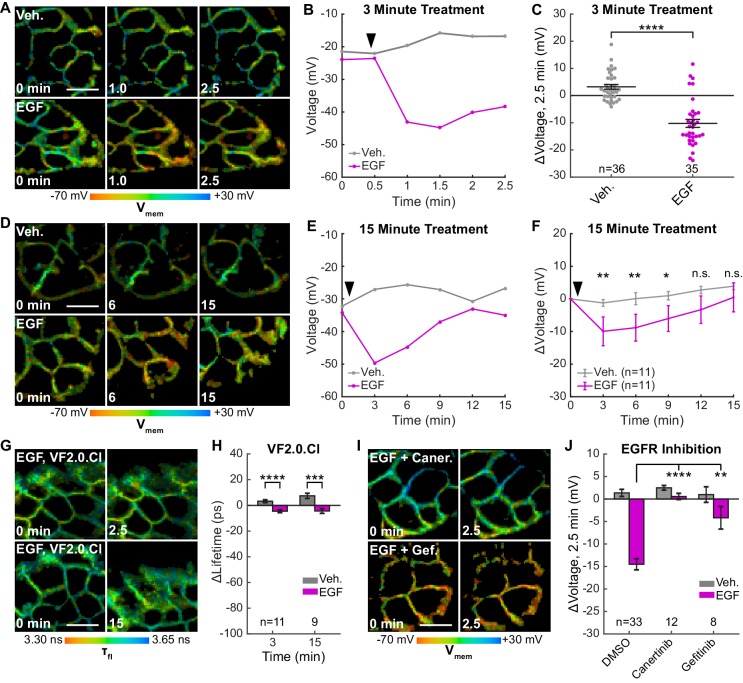
EGFR-mediated receptor tyrosine kinase activity produces a transient hyperpolarization in A431 cells. (**A**) Representative VF-FLIM time series of A431 cells treated with imaging buffer vehicle (top) or 500 ng/mL EGF (80 nM, bottom). (**B**) Quantification of images in (**A**), with Vehicle (Veh.)/EGF added at black arrow. (**C**) Aggregated responses for various trials of cells treated with vehicle or EGF. (**D**) Lifetime images of longer-term effects of vehicle (top) or EGF (bottom) treatment. (**E**) Quantification of images in (**D**). (**F**) Average response of cells over the longer time course. (**G**) Images of VF2.0.Cl (voltage insensitive) lifetime before and after EGF treatment. No τ_fl_ change is observed 2.5 (top) or 15 min (bottom) following EGF treatment. (**H**) Average VF2.0.Cl lifetime changes following EGF treatment. VF2.0.Cl graphs and images are scaled across the same lifetime range (350 ps) as VF2.1.Cl plots and images. The small drift observed would correspond to 2–4 mV of voltage change in VF2.1.Cl lifetime. (**I**) Lifetime images of A431 cells before and after EGF addition, with 500 nM canertinib (top) or 10 μM gefitinib (bottom). (**J**) Voltage changes 2.5 min after EGF addition in cells treated with DMSO (vehicle control) or an EGFR inhibitor. Scale bars are 20 μm. (**C,F,H**): Asterisks indicate significant differences between vehicle and EGF at that time point. (**J**): Asterisks reflect significant differences between EGF-induced voltage responses with DMSO vehicle or an EGFR inhibitor (n.s. p>0.05, *p<0.05, **p<0.01, ***p<0.001, ****p<0.0001, two-tailed, unpaired, unequal variances t-test).

We find that treatment of A431 cells with EGF results in a 15 mV hyperpolarization within 60–90 s in approximately 80% of cells (Figure 4A–C, Figure 4—figure supplement 1, Figure 4—figure supplement 2), followed by a slow return to baseline within 15 min (Figure 4D–F, Figure 4—figure supplements 3 and 0 second acquisitions). The voltage response to EGF is dose-dependent, with an EC_50_ of 90 ng/mL (14 nM) (Figure 4—figure supplement 4). Vehicle-treated cells show very little τ_fl_ change (Figure 4A–F). Identical experiments with voltage-insensitive VF2.0.Cl (Figure 4G–H, Figure 4—figure supplement 1, Figure 4—figure supplement 3, Figure 4—figure supplement 5) reveal little change in τ_fl_ upon EGF treatment, indicating the drop in τ_fl_ arises from membrane hyperpolarization. We observe the greatest hyperpolarization 1 to 3 min after treatment with EGF, which is abolished by inhibition of EGFR and ErbB2 tyrosine kinase activity with the covalent inhibitor canertinib (Figure 4I–J, Figure 4—figure supplement 6). Blockade of the EGFR kinase domain with gefitinib, a non-covalent inhibitor of EGFR, also results in a substantial decrease in the EGF-evoked hyperpolarization (Figure 4I–J, Figure 4—figure supplement 6). Together, these results indicate that A431 cells exhibit an EGF-induced hyperpolarization, which depends on the kinase activity of EGFR and persists on the timescale of minutes.

**Figure 4—figure supplement 1.**
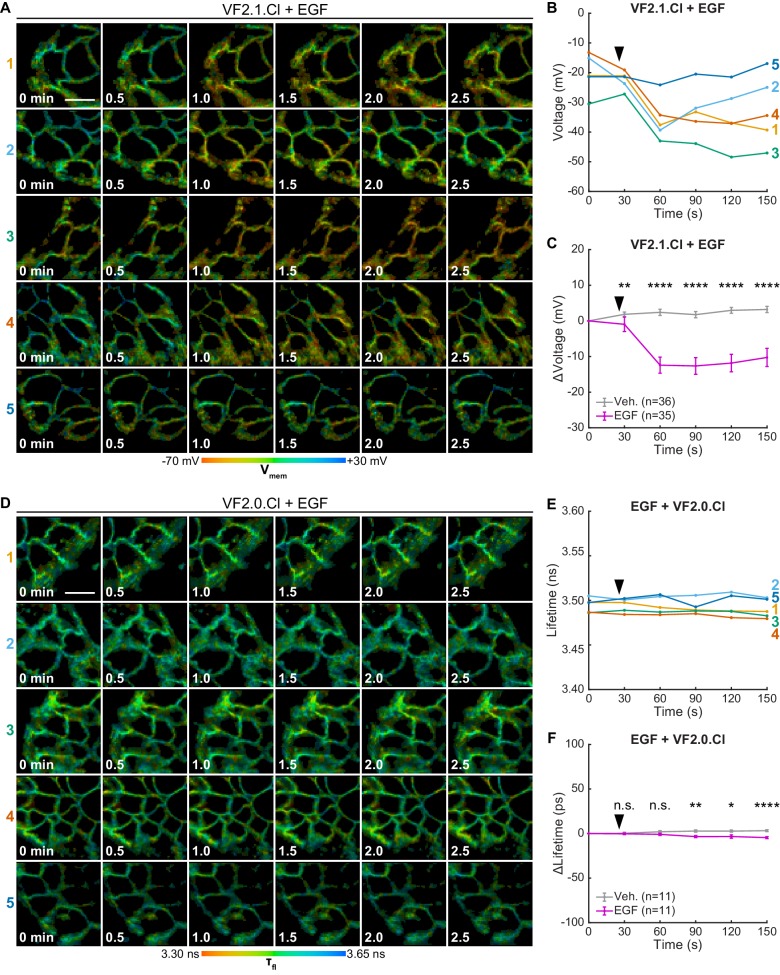
Individual VF-FLIM recordings of A431 EGF response. (**A**) Representative 3 min VF-FLIM recordings of A431 cells loaded with 50 nM VF2.1.Cl. 500 ng/mL EGF was added 30 s into the time series (black arrow). (**B**) Quantification of the images in (**A**), with a single trace per image series shown. (**C**) Average voltage change in A431 cells following the addition of imaging buffer vehicle (gray) or EGF (purple). (**D**) Control VF2.0.Cl (not voltage sensitive, 50 nM) images of A431 cells treated as in (**A**). Images are scaled across the same amount of lifetime space (350 ps) as the VF2.1.Cl images. (**E**) Quantification of the images in (**D**). (**F**) Average VF2.0.Cl lifetime change seen in A431 cells following the addition of imaging buffer vehicle (gray) or EGF (purple) in A431 cells. Graph is scaled across the same amount of lifetime space as the VF2.1.Cl data in (**C**). Asterisks indicate significant differences between vehicle and EGF treated cells at a given time point (n.s. p>0.05, *p<0.05, **p<0.01, ***p<0.001, ****p<0.0001, two-sided, unpaired, unequal variances t-test). Scale bars are 20 μm.

**Figure 4—figure supplement 2.**
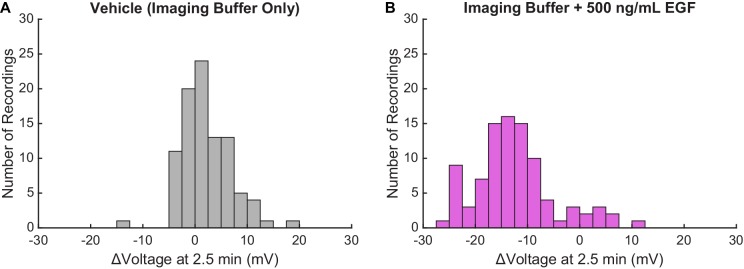
Membrane potential changes in A431 cells 2.5 min after EGF treatment. Comparison of V_mem_ changes observed in A431 cells 2.5 min after treatment with (**A**) imaging buffer vehicle or (**B**) 500 ng/mL EGF. Data shown here are compiled from Figure 4C and Figure 5A to provide a sense of overall distribution of the responses. Each recording contained a single group of approximately 5 to 10 cells. Sample sizes (number of recordings): Vehicle 93, EGF 92.

**Figure 4—figure supplement 3.**
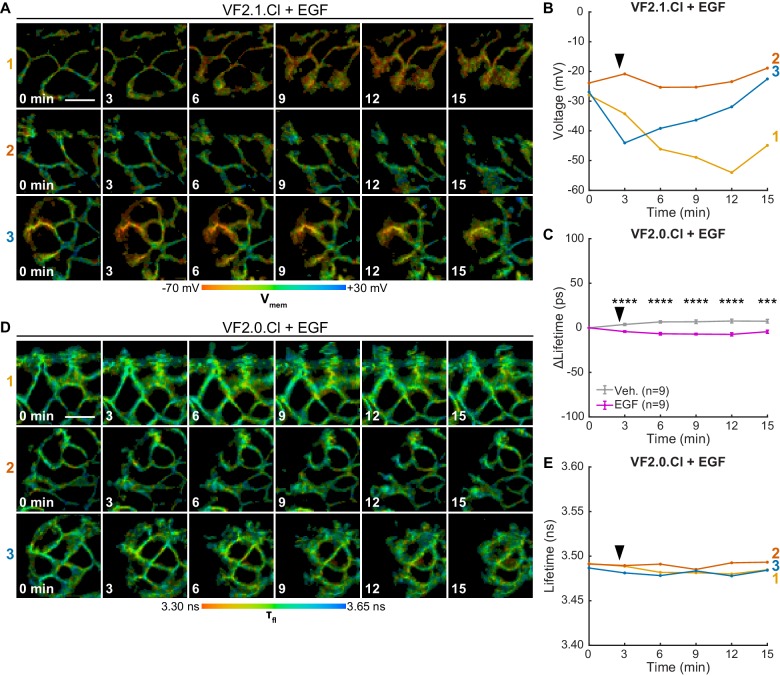
VF-FLIM reports A431 V_mem_ changes over 15 min. (**A**) Representative longer term VF-FLIM recordings of A431 cells loaded with 50 nM VF2.1.Cl. 500 ng/mL EGF was added 30 s into the time series. (**B**) Quantification of the images in (**A**), with a single trace per image series shown. (**C**) Control VF2.0.Cl (not voltage sensitive, 50 nM) images of A431 cells treated as in (**A**). Images are scaled across the same total lifetime range (350 ps) as the VF2.1.Cl images. (**D**) Quantification of the recordings in (**C**). (**E**) Average VF2.0.Cl lifetime change seen in A431 cells following the addition of imaging buffer vehicle (gray) or EGF (purple). Asterisks indicate significant differences between vehicle and EGF treated cells at a given time point (***p<0.001, ****p<0.0001, two-sided, unpaired, unequal variances t-test). Scale bars are 20 μm.

**Figure 4—figure supplement 4.**
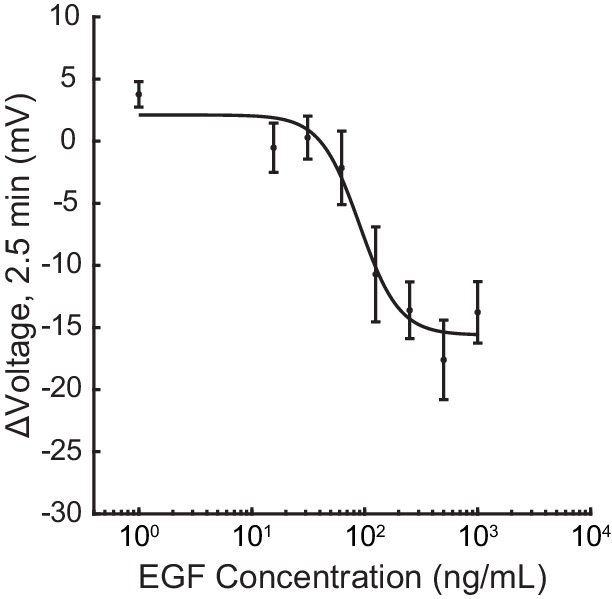
Dose-response relationship of A431 voltage response to EGF. Data were fit to a four-parameter logistic function to obtain an EC_50_ of 90 ng/mL (95% CI: 47–130 ng/mL). Response to each EGF concentration is shown as mean ± SEM of 6 or 7 recordings (one group of 5–10 cells per recording).

**Figure 4—figure supplement 5.**
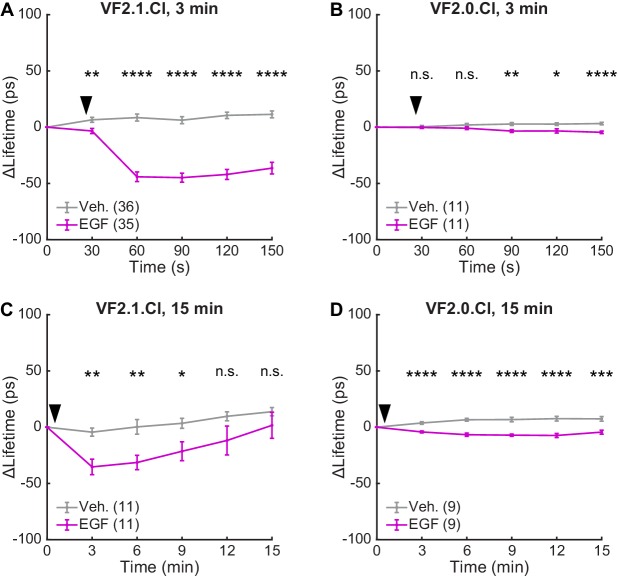
Effect sizes of VF2.1.Cl and VF2.0.Cl response to EGF treatment. Average lifetime changes observed in A431 cells following the addition (black arrow) of imaging buffer vehicle (gray) or 500 ng/mL EGF (purple). (**A**) Cells incubated with 50 nM VF2.1.Cl and imaged for 3 min. (**B**) Cells incubated with 50 nM VF2.0.Cl (not voltage sensitive) and imaged for 3 min. (**C**) Cells incubated with 50 nM VF2.1.Cl and imaged intermittently for 15 min. (**D**) Cells incubated with 50 nM VF2.0.Cl (not voltage sensitive) and imaged intermittently for 15 min. Data are reproduced from Figure 4, Figure 4—figure supplement 1, and Figure 4—figure supplement 3, but here data are scaled in units of lifetime rather than voltage for facile comparison. Data are shown as mean ± SEM for the indicated number of recordings (one group of 5–10 cells per recording). Asterisks indicate significant differences between vehicle and EGF treated cells at a given time point (n.s. p>0.05, *p<0.05, **p<0.01, ***p<0.001, ****p<0.0001, two-sided, unpaired, unequal variances t-test).

**Figure 4—figure supplement 6.**
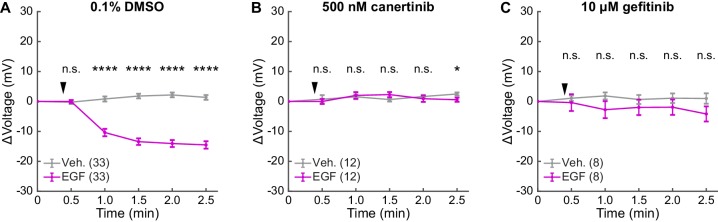
EGFR inhibitors abolish voltage response to EGF in A431 cells. Average V_mem_ changes following the addition (black arrow) of imaging buffer vehicle (gray) or 500 ng/mL EGF (purple) to A431 cells pre-treated with the indicated drug or DMSO vehicle. 2.5 min time points from this data are shown elsewhere (Figure 4J); entire time series are shown here. Data are presented as mean ± SEM for the indicated number of recordings (one group of 5–10 cells per recording). Asterisks indicate significant differences between vehicle and EGF treated cells at a given time point (n.s. p>0.05, *p<0.05, **p<0.01, ***p<0.001, ****p<0.0001, two-sided, unpaired, unequal variances t-test).

**Figure 5.**
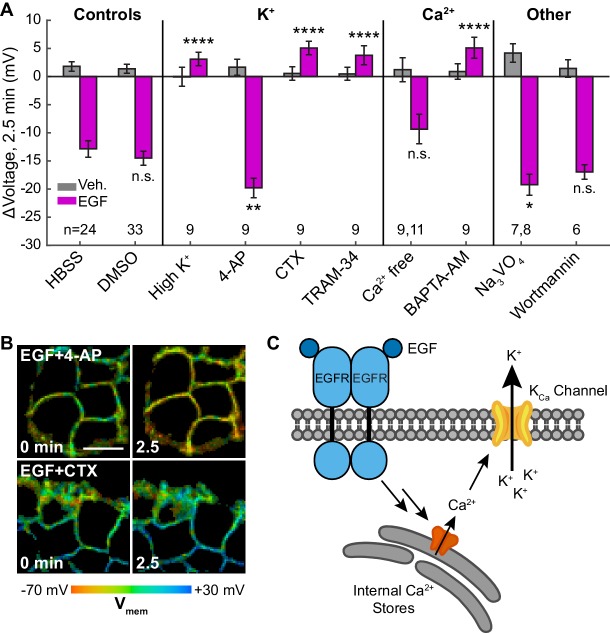
EGF-induced hyperpolarization is mediated by a Ca^2+^ activated K^+^ channel. (**A**) Comparison of the V_mem_ change 2.5 min after EGF addition in cells incubated in unmodified imaging buffer (HBSS) or in modified solutions. (**B**) Lifetime images of A431 cells treated with 4-AP or CTX. (**C**) Model for membrane hyperpolarization following EGFR activation. Scale bar is 20 μm. Bars are mean ± SEM. Sample sizes listed are (Veh, EGF); where only one number is given, sample size was the same for both. Asterisks reflect significant differences in EGF-stimulated V_mem_ change between the unmodified control (HBSS or DMSO) and modified solutions (n.s. p>0.05, *p<0.05, **p<0.01, ***p<0.001, ****p<0.0001, two-tailed, unpaired, unequal variances t-test). DMSO: 0.1% DMSO, high K^+^: 120 mM K^+^, 4-AP: 5 mM 4-aminopyridine, CTX: 100 nM charybdotoxin, TRAM-34: 200 nM TRAM-34, Ca^2+^ free: 0 mM Ca^2+^ and Mg^2+^, BAPTA-AM: 10 μM bisaminophenoxyethanetetraacetic acid acetoxymethyl ester, Na_3_VO_4_: 100 μM sodium orthovanadate, wortmannin: 1 μM wortmannin.

Outward K^+^ currents could mediate EGF-induced hyperpolarization. Consistent with this hypothesis, dissipation of the K^+^ driving force by raising extracellular [K^+^] completely abolishes the typical hyperpolarizing response to EGF and instead results in a small depolarizing potential of approximately 3 mV (Figure 5A, Figure 5—figure supplement 1B). Blockade of voltage-gated K^+^ channels (K_v_) with 4-aminopyridine (4-AP) prior to EGF treatment enhances the hyperpolarizing response to EGF (Figure 5A and B, Figure 5—figure supplement 1C). In contrast, blockade of Ca^2+^-activated K^+^ channels (K_Ca_) with charybdotoxin (CTX) results in a depolarizing potential of approximately 4 mV after exposure to EGF, similar to that observed with high extracellular [K^+^] (Figure 5A and B, Figure 5—figure supplement 1D). TRAM-34, a specific inhibitor of the intermediate-conductance Ca^2+^ activated potassium channel K_Ca_3.1 (Wulff et al., 2000), also abolishes EGF-induced hyperpolarization (Figure 5A, Figure 5—figure supplement 1E). CTX treatment has little effect on the resting membrane potential, while TRAM-34 or 4-AP depolarizes cells by approximately 5–10 mV (Figure 5—figure supplement 2).

**Figure 5—figure supplement 1.**
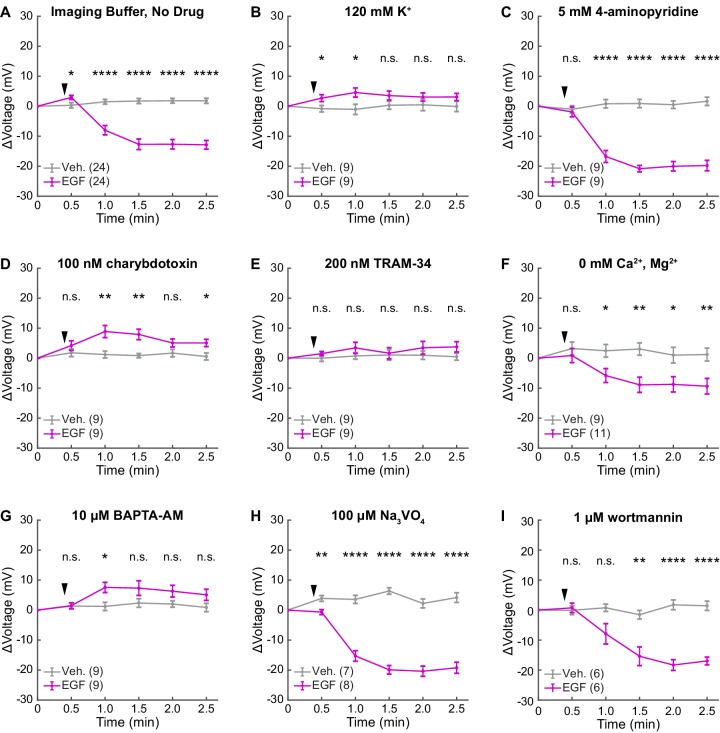
A431 voltage response to EGF with pharmacological intervention. Average V_mem_ changes following the addition (black arrow) of imaging buffer vehicle (gray) or 500 ng/mL EGF (purple) to A431 cells pre-treated with the indicated drug or ionic composition change. 2.5 min time points from this data are shown elsewhere (Figure 5); entire time series are shown here to illustrate the time courses of the large hyperpolarizing current and small depolarizing current. Data are shown as mean ± SEM for the indicated number of recordings (one group of 5–10 cells per recording). Asterisks indicate significant differences between vehicle and EGF treated cells at a given time point (n.s. p>0.05, *p<0.05, **p<0.01, ***p<0.001, ****p<0.0001, two-sided, unpaired, unequal variances t-test).

**Figure 5—figure supplement 2.**
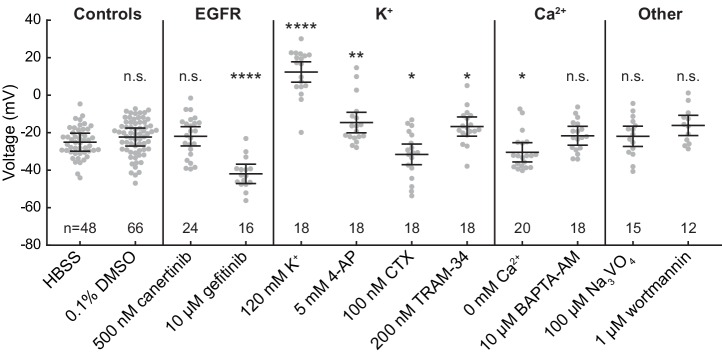
Effects of pharmacological and ionic perturbations on A431 resting membrane potential. Data are the initial V_mem_ reference images for recordings used in EGF addition time series. Data are shown as mean ± SEM for the indicated number of images (one group of 5–10 cells per image), and gray dots represent individual images. Asterisks indicate significant differences between the appropriate vehicle (HBSS or 0.1% DMSO) and pharmacology treated cells (n.s. p>0.05, *p<0.05, **p<0.01, ***p<0.001, ****p<0.0001, two-sided, unpaired, unequal variances t-test). CTX = charybdotoxin, 4-AP = 4-aminopyridine, BAPTA-AM = bisaminophenoxyethanetetraacetic acid acetoxymethyl ester.

To explore the effects of other components of the EGFR pathway on EGF-induced hyperpolarization, we perturbed intra- and extracellular Ca^2+^ concentrations during EGF stimulation. Reduction of extracellular Ca^2+^ concentration did not substantially alter the EGF response (Figure 5A, Figure 5—figure supplement 1F). However, sequestration of intracellular Ca^2+^ with BAPTA-AM disrupts the hyperpolarization response. BAPTA-AM treated cells show a small, 4 mV depolarization in response to EGF treatment, similar to CTX-treated cells (Figure 5A, Figure 5—figure supplement 1G). Perturbation of Ca^2+^ levels had little effect on the resting membrane potential (Figure 5—figure supplement 2). Introduction of wortmannin (1 μM) to block downstream kinase activity has no effect on the membrane potential response to EGF, while orthovanadate addition (Na_3_VO_4_, 100 μM) to block phosphatase activity results in a small increase in the hyperpolarizing response (Figure 5A, Figure 5—figure supplement 1H–I). These results support a model for EGF-EGFR mediated hyperpolarization in which RTK activity of EGFR causes release of internal Ca^2+^ stores to in turn open K_Ca_ channels and hyperpolarize the cell (Figure 5C).

## Discussion

We report the design and implementation of a new method for optically quantifying absolute membrane potential in living cells. VF-FLIM enjoys 100-fold improved throughput over patch clamp electrophysiology, as well as improved spatial resolution. The performance of VF-FLIM hinges on a balance between resolution in three dimensions: membrane potential, space, and time. We discuss the advantages and disadvantages of VF-FLIM in this light, as well as the new application space that is made accessible by VF-FLIM.

### Resolution of VF-FLIM: voltage, space, and time

The key advantage of VF-FLIM over previously reported optical approaches is its superior V_mem_ resolution. Resolution can be interpreted as stability of the τ_fl_-V_mem_ calibration over time and between cells. Any factors other than V_mem_ that change τ_fl_ decrease resolution. VF-FLIM exhibits a 19-fold improvement in inter-cell V_mem_ resolution over FLIM with the GEVI CAESR (Brinks et al., 2015) and a 8-fold improvement over di-8-ANEPPS excitation ratios (Zhang et al., 1998). Although all optical strategies, including VF-FLIM, have worse V_mem_ resolution than modern electrophysiology, the greater throughput, improved spatial resolution, and reduced invasiveness of optical strategies make them a powerful complement to electrode-based recordings.

The sources of variability that reduce resolution of optical V_mem_ measurements are manifold, but two major contributors are membrane specificity of the stain and the complexity of the lipid environment. Nonspecific staining is fluorescence signal from anywhere other than the plasma membrane, such as contaminating intracellular staining from poorly trafficked (CAESR) or internalized (ANEPPS) sensor. In contrast, exogenously loaded VF2.1.Cl exhibits little fluorescence contribution from regions other than the plasma membrane. Secondly, membrane composition and dipole potential can vary between cells and cell lines, changing the local environment of the fluorescent indicator (Wang, 2012; Brügger, 2014). Styryl dyes like di-8-ANEPPS can respond to changes in dipole potential (Zhang et al., 1998; Gross et al., 1994), and VF dyes may be similarly sensitive to dipole potential. Additionally, fluorescence lifetime depends on certain environmental factors (e.g. temperature, viscosity, ionic strength) (Berezin and Achilefu, 2010), which may introduce variability. These parameters are usually determined by the biological system under study, and re-calibration is important if they change dramatically in an experiment.

VF-FLIM, like all optical approaches, improves upon the spatial resolution of patch clamp electrophysiology. While VF-FLIM records the V_mem_ of an optically defined region of interest (in this study a cell or cell group), electrophysiology records V_mem_ at an individual cell or part of a cell where the electrode makes contact, which may or may not reflect the V_mem_ of the entire cell or group. In this study, we interpret VF-FLIM at the whole cell level only, since that is the smallest unit in which the V_mem_ can be reliably calibrated by whole cell patch clamp electrophysiology. Intriguingly, there are differences in lifetime within some cells in VF-FLIM images at the pixel to pixel level. In small, mostly spherical cells under voltage clamp, one would expect uniform membrane potential (Armstrong and Gilly, 1992), so these subcellular differences are most likely noise in the measurement. We speculate that most of this pixel-to-pixel noise comes from variability in fitting the biexponential lifetime model. Lifetime estimates at each pixel are calculated from 20 to 100-fold fewer photons than the lifetime value for the entire ROI. These lower photon counts at the single pixel level produce V_mem_ estimates that are less precise than the V_mem_ estimate for the entire ROI. Collection of more photons at each pixel could likely reduce this noise but would require longer acquisition times. We also cannot fully rule out an alternative explanation that the observed subcellular variability is the result of local differences in membrane composition (Gross et al., 1994).

V_mem_ recordings in systems too large or too small for electrophysiological study could be an important application of VF-FLIM. Despite the improbability of V_mem_ compartmentalization in individual HEK293T cells, other cells with complex morphology and processes may display real, subcellular V_mem_ differences. In addition, delocalized V_mem_ patterns across tissues could in theory be stable (Cervera et al., 2016a) and have been proposed to contribute to tissue development (Levin, 2014). One remaining challenge in expanding VF-FLIM to these areas is the requirement for an initial calibration with voltage clamp electrophysiology. Alternative ways to control V_mem_, such as ionophores or optogenetic actuators (Berndt et al., 2009), may prove useful in these systems. When applying VF-FLIM to tissues, the cellular specificity of the VF stain becomes a consideration, as the VF2.1.Cl indicator used in this study labels all cell membranes efficiently. Looking ahead, recordings in tissue are an exciting area for future development of VF-FLIM, particularly in conjunction with cellular and sub-cellular strategies for targeting VF dyes (Liu et al., 2017; Grenier et al., 2019).

To obtain absolute V_mem_ measurements with fluorescence lifetime, VF-FLIM sacrifices some of the temporal resolution of electrophysiology or intensity-based voltage imaging. VF-FLIM acquisition times are limited by the large numbers of photons needed per pixel in time-correlated single photon counting (see Materials and methods). As a result, VF-FLIM in its current implementation can track V_mem_ events lasting longer than a few seconds. For ‘resting’ membrane potential or V_mem_ dynamics associated with cell growth or differentiation, this temporal resolution is likely sufficient. Nevertheless, in the future, we envision allying VF-FLIM with recently developed, faster lifetime imaging technology to enable optical quantification of more rapid V_mem_ responses (Raspe et al., 2015; Gao et al., 2014).

### Resting membrane potential distributions in cultured cells

Using the improved V_mem_ resolution and throughput of VF-FLIM, we optically documented resting membrane potential distributions in cultured cells to characterize the membrane potential state(s) present. The presence and significance of distinct V_mem_ states in cell populations is mostly uncharacterized due to the throughput limitations of patch-clamp electrophysiology, but some reports suggest that distinct V_mem_ states arise during the various phases of the cell cycle (Ouadid-Ahidouch et al., 2001; Wonderlin et al., 1995). V_mem_ histograms presented in this work appear more or less unimodal, showing no clear sign of cell cycle-related V_mem_ states (Figure 3A,D; Figure 3—figure supplement 1A,D,G). We considered the possibility that VF-FLIM does not detect cell-cycle-related V_mem_ states because we report average V_mem_ across cell groups in cases where cells are in contact (Figure 1—figure supplement 1). This explanation is unlikely for two reasons. First, V_mem_ distributions for CHO cells appear unimodal, even though CHO cultures were mostly comprised of isolated cells under the conditions tested (Figure 3D–F). Second, theoretical work suggests that dramatically different V_mem_ states in adjacent cells are unlikely, as electrical coupling often leads to equilibration of V_mem_ across the cell group (Cervera et al., 2016a; Cervera et al., 2016b). Although we cannot rule out the possibility of poorly separated V_mem_ populations (i.e. with a mean difference in voltage below our resolution limit), VF-FLIM both prompts and enables a re-examination of the notion that bi- or multimodal V_mem_ distributions exist in cultured cells. Furthermore, VF-FLIM represents an exciting opportunity to experimentally visualize theorized V_mem_ patterns in culture and in more complex tissues. Studies towards this end are ongoing in our laboratory.

### Epidermal growth factor induces V_mem _signaling in A431 cells

In the present study, we use VF-FLIM to provide the first cell-resolved, direct visualization of voltage changes induced by growth factor signaling. For long term V_mem_ recordings during growth-related processes, an optical approach is more attractive than an electrode-based one. Electrophysiology becomes increasingly challenging as time scale lengthens, especially if cells migrate, and washout of the cytosol with pipette solution can change the very signals under study (Horn and Korn, 1992; Malinow and Tsien, 1990). Previous attempts to electrophysiologically record V_mem_ in EGF-stimulated A431 cells were unsuccessful due to these technical challenges (Pandiella et al., 1989). Because whole cell voltage-clamp electrophysiology was intractable, the V_mem_ response in EGF-stimulated A431 cells was addressed indirectly through model cell lines expressing EGFR exogenously (Pandiella et al., 1989), bulk measurements on trypsinized cells in suspension (Magni et al., 1991), or cell-attached single channel recordings (Peppelenbosch et al., 1991; Lückhoff and Clapham, 1994; Mozhayeva et al., 1989). By stably recording V_mem_ during EGF stimulation, VF-FLIM enables direct study of V_mem_ signaling in otherwise inaccessible pathways.

In conjunction with physiological manipulations and pharmacological perturbations, we explore the molecular mechanisms underlying EGF-induced hyperpolarization. We find that signaling along the EGF-EGFR axis results in a robust hyperpolarizing current carried by K^+^ ions, passed by the Ca^2+^-activated K^+^ channel K_Ca_3.1, and mediated by intracellular Ca^2+^ (Figure 5C). We achieve a complete loss of the hyperpolarizing response to EGF by altering the K^+^ driving force (‘High K^+^’ Figure 5A, Figure 5—figure supplement 1B), blocking calcium-activated K^+^ currents directly (‘CTX’ and ‘TRAM-34’, Figure 5A, Figure 5—figure supplement 1D,E), or intercepting cytosolic Ca^2+^ (‘BAPTA-AM’, Figure 5A, Figure 5—figure supplement 1G). These results, combined with transcriptomic evidence that K_Ca_3.1 is the major K_Ca_ channel in A431 cells (Thul et al., 2017), indicate that K_Ca_3.1 mediates the observed hyperpolarization. Interestingly, under some conditions where K^+^-mediated hyperpolarization is blocked (‘CTX,’ ‘high K^+'^, ‘BAPTA-AM’), VF-FLIM reveals a small, secondary depolarizing current not visible during normal EGF stimulation. This current likely arises from initial Ca^2+^ entry into the cell, as previously observed during EGF signaling (Pandiella et al., 1987; Marquèze-Pouey et al., 2014). Although we have obtained direct and conclusive evidence of EGF-induced hyperpolarization in A431 cells, the interactions between this voltage change and downstream targets of EGFR remain incompletely characterized. Enhancing EGF signaling by blockade of cellular tyrosine phosphatases with orthovanadate (Reddy et al., 2016) correspondingly increases EGF-mediated hyperpolarization (‘Na_3_VO_4_’ Figure 5A, Figure 5—figure supplement 1H), but inhibition of downstream kinase activity appears to have little effect on hyperpolarization (‘wortmannin’ Figure 5A, Figure 5—figure supplement 1I).

In the context of RTK signaling, V_mem_ may serve to modulate the driving force for external Ca^2+^ entry (Huang and Jan, 2014; Yang and Brackenbury, 2013) and thereby act as a regulator of this canonical signaling ion. Alternatively, V_mem_ may play a more subtle biophysical role, such as potentiating lipid reorganization in the plasma membrane (Zhou et al., 2015). Small changes in V_mem_ likely affect signaling pathways in ways that are currently completely unknown, but high throughput discovery of V_mem_ targets remains challenging. Combination of electrophysiology with single cell transcriptomics has begun to uncover relationships between V_mem_ and other cellular pathways in excitable cells (Cadwell et al., 2016); such approaches could be coupled to higher throughput VF-FLIM methods to explore pathways that interact with V_mem_ in non-excitable contexts.

VF-FLIM represents a novel and general approach for interrogating the roles of membrane potential in fundamental cellular physiology. Future improvements to the voltage resolution could be made by use of more sensitive indicators, which may exhibit larger changes in fluorescence lifetime (Woodford et al., 2015). VF-FLIM can be further expanded to include the entire color palette of PeT-based voltage indicators (Huang et al., 2015; Deal et al., 2016), allied with targeting methods to probe absolute membrane potential in heterogeneous cellular populations (Liu et al., 2017; Grenier et al., 2019), and coupled to high-speed imaging techniques for optical quantification of fast voltage events (Raspe et al., 2015; Gao et al., 2014).

## Materials and methods

**Key resources table:** 

Reagent type (species) or resource	Designation	Source or reference	Identifiers	Additional information
Cell line (*Homo sapiens*, female)	A431	UC Berkeley Cell Culture Facility	RRID:CVCL_0037	Cell line maintained in E. Miller lab
Cell line (*Homo sapiens*, female)	HEK293T	UC Berkeley Cell Culture Facility	RRID:CVCL_0063	Cell line maintained in E. Miller lab
Cell line (*Homo sapiens*, female)	MCF-7	UC Berkeley Cell Culture Facility	RRID:CVCL_0031	Cell line maintained in E. Miller lab
Cell line (*Homo sapiens*, female)	MDA-MB-231	UC Berkeley Cell Culture Facility	RRID:CVCL_0062	Cell line maintained in E. Miller lab
Cell line (*Cricetulus griseus*, female)	CHO	UC Berkeley Cell Culture Facility	RRID:CVCL_0214	Cell line maintained in E. Miller lab
Recombinant DNA reagent	CAESR, FCK-QuasAR2-Citrine	Addgene, PMID: 25118186	Addgene:59172, RRID:Addgene_59172	Developed by Adam Cohen, Harvard University
Peptide, recombinant protein	Recombinant human epidermal growth factor (EGF)	PeproTech	Cat#:AF10015500UG	
Commercial assay or kit	Lipofectamine 3000	Thermo Fisher Scientific	Cat#:L3000008	
Commercial assay or kit	QIAprep spin miniprep kit	VWR International	Cat#:27106	
Chemical compound, drug	Sodium orthovanadate	Sigma-Aldrich	CAS:13721-39-6, Cat#:S6508	Activated before use (Gordon, 1991)
Chemical compound, drug	Canertinib	other	CAS:267243-28-7	Gift from John Kuriyan, UC Berkeley
Chemical compound, drug	Gefitinib	Fisher Scientific	CAS:184475-35-2, Cat#:50-101-6270	
Chemical compound, drug	4-aminopyridine, 4-AP	Sigma-Aldrich	CAS:504-24-5, Cat#:A78403	
Chemical compound, drug	Charybdotoxin, CTX	Sigma-Aldrich	CAS:95751-30-7, Cat#:C7802	
Chemical compound, drug	TRAM-34	Sigma-Aldrich	CAS:289905-88-0, Cat#:T6700	
Chemical compound, drug	BAPTA-AM, bisamino-phenoxy-ethanetetra-acetic acid acetoxymethyl ester	Fisher Scientific	CAS:126150-97-8, Cat#:50-101-0334	
Chemical compound, drug	wortmannin	Fisher Scientific	CAS:19545-26-7, Cat#:ICN19569001	
Software, algorithm	SPCM	Becker and Hickl		
Other	Di-8-ANEPPS	Thermo Fisher Scientific	CAS:157134-53-7, Cat#:D3167	
Other	VF2.1.Cl	Synthesized in-house (Woodford et al., 2015)		
Other	VF2.0.Cl	Synthesized in-house (Woodford et al., 2015)		

VoltageFluor (VF) dyes VF2.1.Cl and VF2.0.Cl were synthesized in house according to previously described syntheses (Woodford et al., 2015). VFs were stored either as solids at room temperature or as 1000x DMSO stocks at −20°C. VF stock concentrations were normalized to the absorption of the dichlorofluorescein dye head via UV-Vis spectroscopy in Dulbecco’s phosphate buffered saline (dPBS, Thermo Fisher Scientific, Waltham, MA) pH 9 with 0.1% sodium dodecyl sulfate (w/v, SDS). Di-8-ANEPPS was purchased from Thermo Fisher Scientific. Di-8-ANEPPS was prepared as a 2 mM (2000x) stock solution in DMSO and stored at −20°C. Di-8-ANEPPS concentrations were determined via UV-Vis spectroscopy in methanol (ε at 498 nm: 41,000 cm^−1^ M^−1^ according to the manufacturer’s certificate of analysis).

All salts and buffers were purchased from either Sigma-Aldrich (St. Louis, MO) or Fisher Scientific. TRAM-34, 4-aminopyridine, and charybdotoxin were purchased from Sigma-Aldrich. Gefitinib, wortmannin, sodium orthovanadate, and BAPTA-AM were purchased from Fisher Scientific. Canertinib was a gift from the Kuriyan laboratory at UC Berkeley. Gefitinib, wortmannin, canertinib, and TRAM-34 were made up as 1000x-10000x stock solutions in DMSO and stored at −20°C. Charybdotoxin was made up as a 1000x solution in water and stored at −80°C. 4-aminopyridine was made up as a 20x stock in imaging buffer (HBSS) and stored at 4°C. Recombinantly expressed epidermal growth factor was purchased from PeproTech (Rocky Hill, NJ) and aliquoted as a 1 mg/mL solution in water at −80°C.

Solid sodium orthovanadate was dissolved in water and activated before use (Gordon, 1991). Briefly, orthovanadate solutions were repeatedly boiled and adjusted to pH 10 until the solution was clear and colorless. 200 mM activated orthovanadate stocks were aliquoted and stored at −20°C.

Unless otherwise noted, all imaging experiments were performed in Hank’s Balanced Salt Solution (HBSS; Gibco/Thermo Fisher Scientific). HBSS composition in mM: 137.9 NaCl, 5.3 KCl, 5.6 D-glucose, 4.2 NaHCO_3_, 1.3 CaCl_2_, 0.49 MgCl_2_, 0.44 KH_2_PO_4_, 0.41 MgSO_4_, 0.34 Na_2_HPO_4_. High K^+^ HBSS was made in-house to 285 mOsmol and pH 7.3, containing (in mM): 120 KCl, 23.3 NaCl, 5.6 D-glucose, 4.2 NaHCO_3_, 1.3 CaCl_2_, 0.49 MgCl_2_, 0.44 KH_2_PO_4_, 0.41 MgSO_4_, 0.34 Na_2_HPO_4_. Nominally Ca^2+^/Mg^2+^ free HBSS (Gibco) contained, in mM: 137.9 NaCl, 5.3 KCl, 5.6 D-glucose, 4.2 NaHCO_3_, 0.44 KH_2_PO_4_, 0.34 Na_2_HPO_4_.

### Methods

#### Cell culture

All cell lines were obtained from the UC Berkeley Cell Culture Facility and discarded after twenty-five passages. A431, HEK293T, MCF-7, and MDA-MB-231 cells were authenticated by short tandem repeat (STR) profiling. All cells were routinely tested for mycoplasma contamination. Cells were maintained in Dulbecco’s Modified Eagle Medium (DMEM) with 4.5 g/L D-glucose supplemented with 10% FBS (Seradigm (VWR); Radnor, PA) and 2 mM GlutaMAX (Gibco) in a 5% CO_2_ incubator at 37°C. Media for MCF-7 cells was supplemented with 1 mM sodium pyruvate (Life Technologies/Thermo Fisher Scientific) and 1x non-essential amino acids (Thermo Fisher Scientific). Media for CHO.K1 (referred to as CHO throughout the text) cells was supplemented with 1x non-essential amino acids. HEK293T and MDA-MB-231 were dissociated with 0.05% Trypsin-EDTA with phenol red (Thermo Fisher Scientific) at 37°C, whereas A431, CHO, and MCF-7 cells were dissociated with 0.25% Trypsin-EDTA with phenol red at 37°C. To avoid potential toxicity of residual trypsin, all cells except for HEK293T were spun down at 250xg or 500xg for 5 min and re-suspended in fresh complete media during passaging.

For use in imaging experiments, cells were plated onto 25 mm diameter poly-D-lysine coated #1.5 glass coverslips (Electron Microscopy Sciences) in six well tissue culture plates (Corning; Corning, NY). To maximize cell attachment, coverslips were treated before use with 1–2 M HCl for 2–5 hr and washed overnight three times with 100% ethanol and three times with deionized water. Coverslips were sterilized by heating to 150°C for 2–3 hr. Before use, coverslips were incubated with poly-D-lysine (Sigma-Aldrich, made as a 0.1 mg/mL solution in phosphate-buffered saline with 10 mM Na_3_BO_4_) for 1–10 hr at 37°C and then washed twice with water and twice with Dulbecco’s phosphate buffered saline (dPBS, Gibco).

A431, CHO, HEK293T, and MCF-7 were seeded onto glass coverslips 16–24 hr before microscopy experiments. MDA-MB-231 cells were seeded 48 hr before use because it facilitated formation of gigaseals during whole-cell voltage clamp electrophysiology. Cell densities used for optical resting membrane potential recordings (in 10^3^ cells per cm^2^) were: A431 42; CHO 42; HEK293T 42; MCF-7 63; MDA-MB-231 42. To ensure the presence of single cells for whole-cell voltage clamp electrophysiology, fast-growing cells were plated more sparsely (approximately 20% confluence) for electrophysiology experiments. Cell densities used for electrophysiology (in 10^3^ cells per cm^2^) were: A431 36–52; CHO 21; HEK293T 21; MCF-7 63; MDA-MB-231 42. To reduce their rapid growth rate, HEK293T cells were seeded onto glass coverslips in reduced glucose (1 g/L) DMEM with 10% FBS, 2 mM GlutaMAX, and 1 mM sodium pyruvate for electrophysiology experiments.

#### Cellular loading of VoltageFluor dyes

Cells were loaded with 1x VoltageFluor in HBSS for 20 min in a 37°C incubator with 5% CO_2_. For most experiments, 100 nM VoltageFluor was used. Serum-starved A431 cells were loaded with 50 nM VoltageFluor. After VF loading, cells were washed once with HBSS and then placed in fresh HBSS for imaging. All imaging experiments were conducted at room temperature under ambient atmosphere. Cells were used immediately after loading the VF dye, and no cells were kept for longer than an hour at room temperature.

#### Whole-cell patch-clamp electrophysiology

Pipettes were pulled from borosilicate glass with filament (Sutter Instruments, Novato, CA) with resistances ranging from 4 to 7 MΩ with a P97 pipette puller (Sutter Instruments). Internal solution composition, in mM (pH 7.25, 285 mOsmol/L): 125 potassium gluconate, 10 KCl, 5 NaCl, 1 EGTA, 10 HEPES, 2 ATP sodium salt, 0.3 GTP sodium salt. EGTA (tetraacid form) was prepared as a stock solution in either 1 M KOH or 10 M NaOH before addition to the internal solution. Pipettes were positioned with an MP-225 micromanipulator (Sutter Instruments). A liquid junction potential of −14 mV was determined by the Liquid Junction Potential Calculator in the pClamp software package (Barry, 1994) (Molecular Devices, San Jose, CA), and all voltage step protocols were corrected for this offset.

For VF-FLIM and CAESR, electrophysiology recordings for VF-FLIM and CAESR were made with an Axopatch 200B amplifier and digitized with a Digidata 1440A (Molecular Devices). The software package used was pClamp 10.3. Signals were filtered with a 5 kHz low-pass Bessel filter. Correction for pipette capacitance was performed in the cell attached configuration. Voltage-lifetime calibrations were performed in V-clamp mode, with the cell held at the potential of interest for 15 or 30 s while lifetime was recorded. Potentials were applied in random order, and membrane test was conducted between each step to verify the quality of the patch. For single cell patching, recordings were only included if they maintained a 30:1 ratio of membrane resistance (R_m_) to access resistance (R_a_) and an R_a_ value below 30 MΩ throughout the recording. Due to the reduced health of HEK293T cells transfected with CAESR, recordings were used as long as they maintained a 10:1 R_m_:R_a_ ratio, although most recordings were better than 30:1 R_m_:R_a_. Only recordings stable for at least four voltage steps (roughly 2 min) were included in the dataset.

For di-8-ANEPPS, electrophysiology recordings were made in the same manner as the above, with the following minor differences. Signals were digitized with a Digidata 1550B; the pClamp 10.6 software package was used (Molecular Devices). Potentials were applied in the order 0 mV, −80 mV, +40 mV, −40 mV, +80 mV for ten seconds at each step. Patch parameters were tested at the beginning and end of the patch program, rather than between each step. Only patches that retained a 30:1 ratio of Rm to Ra and access resistance below 30 MΩ throughout the recording were included in the dataset.

For electrophysiology involving small groups of cells (Figure 2—figure supplement 3), complete voltage clamp across the entire cell group was not possible. Recordings were used as long as R_a_ remained below 30 MΩ for at least three voltage steps. Most recordings also retained R_m_:R_a_ ratios greater than 20:1.

#### Epidermal growth factor treatment

A431 cells were serum starved prior to epidermal growth factor studies. Two days before the experiment, cells were trypsizined and suspended in complete media with 10% FBS. Cells were then spun down for 5 min at 500xg and re-suspended in reduced serum DMEM (2% FBS, 2 mM GlutaMAX, 4.5 g/L glucose). Cells were seeded onto 25 mm coverslips in six well plates at a density of 84 × 10^3^ cells per cm^2^. 4–5.5 hr before the experiment, the media was exchanged for serum-free DMEM (0% FBS, 2 mM GlutaMAX, 4.5 g/L glucose).

After 4–5.5 hr in serum-free media, cells were loaded with 50 nM VF dye as described above. In pharmacology experiments, the drug or vehicle was also added to the VF dye loading solution. All subsequent wash and imaging solutions also contained the drug or vehicle. For changes to buffer ionic composition, VoltageFluor dyes were loaded in unmodified HBSS to avoid toxicity from prolonged incubation with high K^+^ or without Ca^2+^. Immediately prior to use, cells were washed in the modified HBSS (120 mM K^+^ or 0 mM Ca^2+^) and recordings were made in the modified HBSS.

For analysis of short-term responses to EGF (3 min time series), VF lifetime was recorded in 6 sequential 30 s exposures. Immediately after the conclusion of the first frame (30–35 s into the recording), EGF or vehicle (imaging buffer only) was added to the indicated final concentration from a 2x solution in HBSS imaging buffer. For analysis of long-term responses to EGF (15 min time series), EGF addition occurred in the same way, but a gap of 150 s (without laser illumination) was allotted between each 30 s lifetime recording. Times given throughout the text correspond to the start of an exposure. Voltage changes at 2.5 min were calculated from the difference between an initial image (taken before imaging buffer vehicle or EGF addition) and a final image (a 30 s exposure starting 2.5 min into the time series).

#### Transfection and imaging of CAESR in HEK293T

The CAESR plasmid was obtained as an agar stab (FCK-Quasar2-Citrine, Addgene #59172), cultured overnight in LB with 100 μg/mL ampicillin, and isolated via a spin miniprep kit (Qiagen). HEK293T cells were plated at a density of 42,000 cells per cm^2^directly onto a six well tissue culture plate and incubated at 37°C in a humidified incubator for 24 hr prior to transfection. Transfections were performed with Lipofectamine 3000 according to the manufacturer’s protocol (Thermo Fisher Scientific). Cells were allowed to grow an additional 24 hr after transfection before they were plated onto glass coverslips for microscopy experiments (as described above for electrophysiology of untransfected HEK293T cells).

#### Determination of EC_50_ for EGF in A431 cells

Average voltage changes 2.5 min after addition of EGF to serum deprived A431 cells were determined at different EGF concentrations, and these means were fit to a four parameter logistic function in MATLAB (MathWorks, Natick, MA).

#### Goldman-Hodgkin-Katz estimation of V_mem _ranges in different imaging buffers

If intracellular and extracellular concentrations, as well as relative permeabilities, of all ionic species are known, the Goldman-Hodgkin-Katz (GHK) equation (Equation 1) can be used to calculate the resting membrane potential of a cell (Hodgkin and Katz, 1949). In practice, the intracellular ion concentrations [X]_in_ and relative permeabilities P_x_ are difficult to determine, so the GHK equation is not a substitute for direct measurement of V_mem_. To obtain a range of reasonable V_mem_ values in systems where these concentrations and relative permeabilities are not known, we calculated possible V_mem_ using the ‘standard’ parameters derived from Hodgkin and Katz (1949), as well as a value above and a value below each ‘standard’ point. The values evaluated were the following: P_K_ 1; P_Na_ 0.01, 0.05, 0.2; P_Cl_ 0.2, 0.45, 0.9; [K^+^]_in_ 90, 150, 200 mM; [Na^+^]_in_ 5, 15, 50 mM; [Cl^-^]_in_ 2, 10, 35 mM. Extracellular ion concentrations [X]_out_ were known (see Materials and methods). In Equation 1, R is the universal gas constant, T is the temperature (293 K for this experiment), and F is Faraday’s constant.

(1) $$V_{mem}=\frac{RT}{F}ln\frac{P_{K}{\left[K^{+}\right]}_{out}+P_{Na}{\left[{Na}^{+}\right]}_{out}+P_{Cl}{\left[{Cl}^{-}\right]}_{in}}{P_{K}{\left[K^{+}\right]}_{in}+P_{Na}{\left[{Na}^{+}\right]}_{in}+P_{Cl}{\left[{Cl}^{-}\right]}_{out}}$$

#### Fluorescence lifetime data acquisition

Fluorescence lifetime imaging was conducted on a LSM 510 inverted scanning confocal microscope (Carl Zeiss AG, Oberkochen, Germany) equipped with an SPC-150 or SPC-150N single photon counting card (Becker and Hickl GmbH, Berlin, Germany) (Scheme 1). 80 MHz pulsed excitation was supplied by a Ti:Sapphire laser (MaiTai HP; SpectraPhysics, Santa Clara, CA) tuned to 958 nm and frequency-doubled to 479 nm. The laser was cooled by a recirculating water chiller (Neslab KMC100). Excitation light was directed into the microscope with a series of silver mirrors (Thorlabs, Newton, NJ or Newport Corporation, Irvine, CA).

Excitation light power at the sample was controlled with a neutral density (ND) wheel and a polarizer (P) followed by a polarizing beamsplitter (BS). Light was titrated such that VoltageFluor lifetime did not drift during the experiment, no phototoxicity was visible, and photon pile-up was not visible on the detector. For recordings at high VoltageFluor concentrations (Figure 1—figure supplement 2, Figure 2—figure supplement 4), reduced power was used to avoid saturating the detector. For optical voltage determinations using 50 or 100 nM VoltageFluor, typical average power at the sample was 5 μW.

Fluorescence emission was collected through a 40x oil immersion objective (Zeiss) coated with immersion oil (Immersol 518F, Zeiss). Emitted photons were detected with a hybrid detector, HPM-100–40 (Becker and Hickl), based on a Hamamatsu R10467 GaAsP hybrid photomultiplier tube. Detector dark counts were kept below 1000 per second during acquisition. Emission light was collected through a 550/49 bandpass filter (Semrock, Rochester, NY) after passing through a 488 LP dichroic mirror (Zeiss). The reference photons for determination of photon arrival times were detected with a PHD-400-N high speed photodiode (Becker and Hickl). Data were acquired with 256 time bins in the analog-to-digital-converter and either 64 × 64 or 256 × 256 pixels of spatial resolution (see discussion of pixel size below).

Routine evaluation of the proper functioning of the lifetime recording setup was performed by measurement of three standards (Figure 1—source data 2): 2 μM fluorescein in 0.1 N NaOH, 1 mg/mL erythrosin B in water (pH 7), and the instrument response function (IRF). The IRF was determined from a solution of 500 μM fluorescein and 12.2 M sodium iodide in 0.1 N NaOH. Because of the high concentration of iodide quencher, the IRF solution has a lifetime shorter than the detector response time, allowing approximation of the instrument response function under identical excitation and emission conditions as data acquisition (Liu et al., 2014).

#### IRF deconvolution

Signal from photons detected in a TCSPC apparatus are convolved with the instrument response (IRF). IRFs can be approximated by the SPCImage fitting software, but consistency of lifetime fits on VF-FLIM datasets was improved by using a measured IRF. Measured IRFs were incorporated by the iterative reconvolution method using SPCImage analysis software (Becker, 2012).

#### VoltageFluor lifetime fitting model

All VoltageFluor lifetime data were fit using SPCImage (Becker and Hickl), which solves the nonlinear least squares problem using the Levenberg-Marquadt algorithm. VF2.1.Cl lifetime data were fit to a sum of two exponential decay components (Equation 2). Attempts to fit the VF2.1.Cl data with a single exponential decay (Equation 3) were unsatisfactory.

(2) Ft=a1e-tτ1+a2e-tτ2

The fluorescence lifetime of VF2.0.Cl was adequately described by a single exponential decay for almost all data (Equation 3). A second exponential component was necessary to fit data at VF2.0.Cl concentrations above 500 nM, likely attributable to the concentration-dependent decrease in lifetime that was observed high VF concentrations.

(3) Ft=ae-tτ

For all data fit with the two component model, the weighted average of the two lifetimes, τ_m_ (Equation 4), was used in subsequent analysis.

(4) $$\tau_{m}=\frac{a_{1}\tau_{1}+a_{2}\tau_{2}}{a_{1}+a_{2}}$$

All lifetime images are represented as an overlay of photon count (pixel intensity) and weighted average lifetime (pixel color) throughout the text (τ_m_ + PC, Figure 1—figure supplement 1). Pixels with insufficient signal to fit a fluorescence decay are shown in black. The photon counts, as well as the lifetimes, in image sequences on the same set of cells are scaled across the same range.

#### Additional fit parameters for VoltageFluor lifetimes

Pixels with photon counts below 300 (VF2.1.Cl) or 150 (VF2.0.Cl) photons at the peak of the decay (time bin with the most signal) were omitted from analysis to ensure reproducible fits. Because the lifetime of VFs does not fully decay to baseline in a single 12.5 ns laser cycle, the incomplete multiexponentials fitting option was used, allowing the model to attribute some signal early in the decay to the previous laser cycle. Out of 256 time bins from the analog-to-digital converter (ADC), only data from time bins 23 to 240 were used in the final fit. The offset parameter (detector dark counts per ADC time bin per pixel) was set to zero. The number of iterations for the fit in SPCImage was increased to 20 to obtain converged fits. Shift between the IRF and the decay trace was fixed to 0.5 (in units of ADC time bins), which consistently gave lifetimes of standards erythrosin B (1 mg/mL in H_2_O) (Boens et al., 2007) and fluorescein (2 μM in 0.1 N NaOH, H_2_O) (Magde et al., 1999) closest to reported values (Figure 1—source data 2).

#### Acquisition time and effective pixel size in lifetime data

To obtain sufficient photons but keep excitation light power minimal, binning between neighboring pixels was employed during fitting. This procedure effectively takes the lifetime as a spatial moving average across the image by including adjacent pixels in the decay for a given pixel. To obtain larger photon counts, the confocal pinhole was set between 2.5 and 3.5 airy units, which corresponds to optical section thickness of approximately 2.5 µm.

**Table:** 

Data type	Acquired pixel width (μm)	Binned pixel width (μm)	Acquisition time (s)	Img size (pixels)
Concentration Curve (Figure 1—figure supplement 2, Figure 2—figure supplement 4)	0.44	3.08	75–90	256 × 256
V_mem_ Distributions (Figure 3)	1.24	8.68	90–120	256 × 256
Electrophysiology Recording	1.00	3.01	15–30	64 × 64
EGF Time Series	0.88	2.64	30	64 × 64

All tabulated values are for an individual frame, although multiple sequential frames were recorded in both the electrophysiology and EGF experiments. For each recording type, the width of each pixel at acquisition is reported, as well as the width of the area included in the binned lifetime signal during fitting. All pixels are square. The acquisition time reflects the total time to collect the image, not the total time exposing each pixel. All FLIM images have 256 time bins in the ns regime, so a 256 × 256 spatial image size represents a 256 × 256 × 256 total dataset. Img = image.

#### Determination of regions of interest

Images were divided into cell groups, with each cell group as a single region of interest (ROI). ROIs were determined from photon count images, either manually from the cell morphology in FIJI (Schindelin et al., 2012) or automatically by sharpening and then thresholding the signal intensity with custom MATLAB code (Source code 2). Regions of images that were partially out of the optical section or contained punctate debris were omitted. Sample ROIs are shown in Figure 1—figure supplement 1.

For cells that adjoin other cells, attribution of a membrane region to one cell versus the other is not possible. As such, we chose to interpret each cell group as an independent sample (‘n’) instead of extracting V_mem_ values for individual cells. Adjacent cells in a group are electrically coupled to varying degrees, and their resting membrane potentials are therefore not independent (Meşe et al., 2007). While this approach did not fully utilize the spatial resolution of VF-FLIM, it prevented overestimation of biological sample size for the effect in question.

#### Conversion of lifetime to transmembrane potential

The mean τ_m_ across all pixels in an ROI was used as the lifetime for that ROI. Lifetime values were mapped to transmembrane potential via the lifetime-V_mem_ standard curves determined with whole-cell voltage-clamp electrophysiology. For electrophysiology measurements, the relationship between the weighted average lifetime (Equation 4) and membrane potential for each patched cell was determined by linear regression, yielding a sensitivity (*m,* ps/mV) and a 0 mV lifetime (*b*, ps) for each cell (Equation 5). The average sensitivity and 0 mV point across all cells of a given type were used to convert subsequent lifetime measurements (τ) to V_mem_ (Figure 2—source data 1, Equation 6). For quantifying changes in voltage (ΔV_mem_) from changes in lifetime (Δτ), only the average sensitivity is necessary (Equation 7).

(5) $$\tau =m*V_{mem}+b$$

(6) $$V_{mem}=\frac{\left(\tau -b\right)}{m}$$

(7) $$\Delta V_{mem}=\frac{\left(\Delta \tau \right)}{m}$$

Where standard error of the mean of a voltage determination (δV_mem_) is given, error was propagated to include the standard errors of the slope (δ*m*) and y-intercept (δ*b*) of the voltage calibration, as well as the standard error of the lifetime measurements (δτ) in the condition of interest (Equation 8). For error in a voltage change (δΔV_mem_), only error in the calibration slope was included in the propagated error (Equation 9). Where standard deviation of VF-FLIM derived V_mem_ values is shown, a similar error propagation procedure was applied, using the standard deviation of the average sensitivity and 0 mV lifetime for that cell line.

(8) $$\delta V_{mem}=\left|V_{mem}\right|\sqrt{{\left(\frac{\sqrt{{\delta \tau }^{2}+{\delta b}^{2}}}{\tau -b}\right)}^{2}+{\left(\frac{\delta m}{m}\right)}^{2}}$$

(9) $$\delta \Delta V_{mem}=\left|\Delta V_{mem}\right|\sqrt{{\left(\frac{\delta \Delta \tau }{\Delta \tau }\right)}^{2}+{\left(\frac{\delta m}{m}\right)}^{2}}$$

#### Resolution of VF-FLIM voltage determination

The intrinsic nature of fluorescence lifetime introduces a point of reference into the voltage measurement, from which a single lifetime image can be interpreted as resting membrane potential. Differences in this reference point (reported here as the 0 mV lifetime) over time and across cells provides an estimate of the voltage-independent noise in VF-FLIM. We report resolution as the root-mean-square deviation (RMSD) between the optically calculated voltage (V_FLIM_) and the voltage set by whole-cell voltage clamp (V_ephys_), which is analogous to the resolution calculations described previously by Cohen and co-workers (Hou et al., 2014). The RMSD of n measurements (Equation 10) can be determined from the variance σ (Cone and Cone, 1976) (Equation 11) and the bias (Equation 12) of the estimator (in this case, VF-FLIM) relative to the ‘true’ value (in this case, electrophysiology). These calculations are described graphically in Scheme 2 below.

(10) $$RMSD=\sqrt{\sigma^{2}+{Bias}^{2}}$$

(11) $$\sigma^{2}=\frac{1}{n}\sum_{i=1}^{n}{\left(V_{FLIM,i}-V_{ephys,i}\right)}^{2}$$

(12) $$Bias=\frac{1}{n}\sum_{i=1}^{n}V_{FLIM,i}-\frac{1}{n}\sum_{i=1}^{n}V_{ephys,i}$$

The voltage-independent variations in lifetime are much larger between cells than within a cell. Therefore, the error in measuring absolute voltage *changes* on a given cell (‘intra-cell’ comparisons) is lower than the error in determining the absolute V_mem_ of that cell (‘inter-cell’ comparisons, since the calibration used is from another cell). We can therefore determine an ‘intra-cell’ RMSD and an ‘inter-cell’ RMSD to reflect the voltage resolution of these two types of measurements. To calculate ‘intra-cell’ error, we look at the RMSD between V_ephys_ and V_FLIM_ using the τ_fl_-V_mem_ relationship *for that specific cell*. Phrased another way, we are looking at the amount of error that would be expected in estimating V_mem_ of a cell if its exact τ_fl_-V_mem_ relationship were known. This ‘intra cell’ RMSD estimates the error expected in quantifying changes in V_mem_ on a given cell. We calculate an intra cell error for each cellular recording, so intra cell errors are reported throughout the text as a mean ± SEM of the intra cell errors for all individual cells of a given type. The average intra cell error was at or below 5 mV for all cell lines tested (Figure 2—source data 1).

The error in the absolute membrane potential determination (‘inter-cell’) is calculated here as the RMSD between the y-intercept (0 mV lifetime) of all of the individual cells’ lifetime-voltage relationships and the 0 mV value for the averaged calibration *for all cells of a given type*. This metric quantifies how well the lifetime-V_mem_ relationship for a given cell line represents an individual cell’s lifetime-V_mem_ relationship. This ‘inter cell’ RMSD ranged from 10 to 23 mV for the tested cell lines (Figure 2—source data 1). Much smaller errors for a population value of V_mem_ can be obtained by averaging V_mem_ recordings from multiple cells.

This method of calculating error assumes that the electrophysiology measurement is perfectly accurate and precise. Realistically, it is likely that some of the variation seen is due to the quality of the voltage clamp. As a result, these RMSD values provide a conservative upper bound for the voltage errors in VF-FLIM.

#### Analysis of CAESR lifetimes

For sample images of CAESR in HEK293T (Figure 1—figure supplement 4), fluorescence decays were fit using SPCImage to a biexponential decay model as described for VF2.1.Cl above, using a peak photon threshold of 150 and a bin of 2 (binned pixel width of 5 μm). To better match the studies by Cohen and co-workers (Brinks et al., 2015), which isolated the membrane fluorescence from cytosolic fluorescence by directing the laser path, the lifetime-voltage relationships were not determined with these square-binned images. Instead, membranes were manually identified, and the fluorescence decays from all membrane pixels were summed together before fitting once per cell. (This is in contrast to the processing of VoltageFluor data, where the superior signal to noise and localization enables fitting and analysis of the lifetime on a pixel by pixel basis). This ‘one fit per membrane’ analysis of CAESR was performed in custom MATLAB code implementing a Nelder-Mead algorithm (Source code 1, adapted from Enderlein and Erdmann, 1997). CAESR data were fit to a biexponential model with the offset fixed to 0 and the color shift as a free parameter.

#### Di-8-ANEPPS ratio-based imaging

In preparation for imaging, HEK293T cells were plated as described above for electrophysiology. 1 µM di-8-ANEPPS was loaded for ten minutes in HBSS at room temperature and atmospheric CO_2_. Coverslips were washed twice in HBSS and transferred to fresh HBSS for imaging. No surfactants were used in the loading (e.g. Pluronic F-127) because their presence worsened cell robustness for whole-cell patch-clamp electrophysiology. All recordings were made with HBSS as an extracellular solution; no cells were kept for more than 30 min after dye loading due to the increasing presence of internalized dye.

Epifluorescence imaging was performed with an inverted Observer.Z1 (Carl Zeiss Microscopy) controlled with µManager 1.4 (Open Imaging) (Edelstein et al., 2014). Images were acquired with an Orca Flash 4 Digital CMOS camera (Hamamatsu Corporation; San Jose, CA). Excitation light was provided with a Spectra X light engine (Lumencor, Inc.; Beaverton, OR). Excitation wavelengths were selected with built-in filters in the Spectra X (440/20 bandpass filter for blue and 550/15 bandpass filter for green). Blue-excited images were obtained with an excitation power of 71 mW/mm^2^ and an exposure time of 50 ms. Green-excited images were obtained with an excitation power of 136 mW/mm^2^ and an exposure time of 500 ms. Emission light was collected with a 40x magnification oil immersion objective lens using Immersol 518F immersion oil (Zeiss). Fluorescence emission was selected with a 562 nm long pass dichroic mirror and further filtered by a 593/40 bandpass filter (Semrock). Excitation and emission wavelengths were selected to match previous work with this probe as closely as possible (Zhang et al., 1998) (current excitation [blue]: 440 ± 10 nm; reported excitation [blue]: 440 ± 15 nm; current excitation [green]: 550 ± 7.5 nm; reported excitation [green]: 530 ± 15 nm; current dichroic: 562 nm long-pass; reported dichroic: 565 nm; current emission: 593 ± 20 nm; reported emission: 570 nm long pass).

#### Di-8-ANEPPS data analysis

Single color (e.g. blue excited or green excited) fluorescence images were background subtracted at each pixel before ratios were calculated. The background value was determined from a region of interest near the center of the image that contained no cells and minimal fluorescent debris. Excitation ratios (‘R’, blue signal divided by green signal, B/G) were then calculated pixelwise from the background subtracted fluorescence images. Pixels with less than 100 arbitrary units of signal in either the blue or the green channel were excluded from analysis and are depicted in black. Regions of interest (ROIs) were manually selected in FIJI to include only area corresponding to the cell membrane. The ratio was averaged across all pixels in a given ROI (similar to the treatment for VF-FLIM, as described in Figure 1—figure supplement 1). The ratio values per value of V_mem_ (set by whole cell patch clamp electrophysiology) in Figure 1—figure supplement 5E,F are the average of these cell-averaged ratios obtained in 6 or 7 sequential images acquired while the V_mem_ was held at the indicated value.

Where normalized R values are discussed, these values were calculated by dividing the ratio at a given potential (averaged for an ROI as discussed above) by the ratio at 0 mV, as reported previously (Zhang et al., 1998). This normalization procedure requires electrode-based calibration for every individual recording and cannot be stably extended to all cells from a particular cell line. Therefore, it is not analogous to VF-FLIM and is not the point of comparison for voltage resolution.

#### Statistical analysis

Mean ± standard error of the mean (SEM) of data is reported throughout the text. Hypothesis testing was performed as indicated with either analysis of variance (ANOVA) followed by appropriate post hoc tests or two-sided, unpaired, unequal variances t-tests. Statistical tests were performed in Python 2 or 3 with the SciPy, pandas and Pingouin (Vallat, 2018) packages. Unless otherwise noted, all data shown reflect at least three biological replicates (independent cultures measured on different days). Each of these biological replicates contained between 1 and 5 technical replicates (different samples of cells that were measured on the same day and had been prepared from the same cell stock). For tandem electrophysiology-FLIM measurements, each τ_fl_-V_mem_ calibration includes at least three biological replicates to capture the variability expected during applications of VF-FLIM. No power analyses were performed before data were collected. Sample sizes throughout the text refer to the total number of cells or cell groups of a given type analyzed across all biological and technical replicates. Cell group identification is discussed in Methods. For experiments where resting membrane potential or resting membrane potential changes are compared to a baseline (Figures 3–5 and supplements), both control measurements and their physiologically or pharmacologically altered counterparts were recorded on each experimental day. Masking was not used during data collection or analysis.

## Data Availability

All data presented in the manuscript is available in the supporting/supplementary information.

## Additional files

Source code 1. Global analysis of CAESR fluorescence lifetimes.

Source code 2. Automated thresholding and cell group identification.

Transparent reporting form

## References

[bib1] Abdul Kadir L, Stacey M, Barrett-Jolley R (2018). Emerging roles of the membrane potential: action beyond the action potential. Frontiers in Physiology.

[bib2] Adams DS, Levin M (2012). General principles for measuring resting membrane potential and ion concentration using fluorescent bioelectricity reporters. Cold Spring Harbor Protocols.

[bib3] Armstrong CM, Gilly WF (1992). Access resistance and space clamp problems associated with whole-cell patch clamping. Methods in Enzymology.

[bib4] Barry PH (1994). JPCalc, a software package for calculating liquid junction potential corrections in patch-clamp, intracellular, epithelial and bilayer measurements and for correcting junction potential measurements. Journal of Neuroscience Methods.

[bib5] Becker W (2012). The bh TCSPC Handbook.

[bib6] Berezin MY, Achilefu S (2010). Fluorescence lifetime measurements and biological imaging. Chemical Reviews.

[bib7] Berndt A, Yizhar O, Gunaydin LA, Hegemann P, Deisseroth K (2009). Bi-stable neural state switches. Nature Neuroscience.

[bib8] Blacker TS, Duchen MR (2016). Investigating mitochondrial redox state using NADH and NADPH autofluorescence. Free Radical Biology and Medicine.

[bib9] Boens N, Qin W, Basarić N, Hofkens J, Ameloot M, Pouget J, Lefèvre JP, Valeur B, Gratton E, vandeVen M, Silva ND, Engelborghs Y, Willaert K, Sillen A, Rumbles G, Phillips D, Visser AJ, van Hoek A, Lakowicz JR, Malak H, Gryczynski I, Szabo AG, Krajcarski DT, Tamai N, Miura A (2007). Fluorescence lifetime standards for time and frequency domain fluorescence spectroscopy. Analytical Chemistry.

[bib10] Briggman KL, Kristan WB, González JE, Kleinfeld D, Tsien RY (2010). Monitoring integrated activity of individual neurons using FRET-based voltage-sensitive dyes. Membrane Potential Imaging in the Nervous System: Methods and Applications.

[bib11] Brinks D, Klein AJ, Cohen AE (2015). Two-photon lifetime imaging of voltage indicating proteins as a probe of absolute membrane voltage. Biophysical Journal.

[bib12] Brügger B (2014). Lipidomics: analysis of the lipid composition of cells and subcellular organelles by electrospray ionization mass spectrometry. Annual Review of Biochemistry.

[bib13] Bullen A, Saggau P (1999). High-speed, random-access fluorescence microscopy: ii. fast quantitative measurements with voltage-sensitive dyes. Biophysical Journal.

[bib14] Cadwell CR, Palasantza A, Jiang X, Berens P, Deng Q, Yilmaz M, Reimer J, Shen S, Bethge M, Tolias KF, Sandberg R, Tolias AS (2016). Electrophysiological, transcriptomic and morphologic profiling of single neurons using Patch-seq. Nature Biotechnology.

[bib15] Cervera J, Alcaraz A, Mafe S (2016a). Bioelectrical signals and ion channels in the modeling of multicellular patterns and cancer biophysics. Scientific Reports.

[bib16] Cervera J, Meseguer S, Mafe S (2016b). The interplay between genetic and bioelectrical signaling permits a spatial regionalisation of membrane potentials in model multicellular ensembles. Scientific Reports.

[bib17] Chen RF, Knutson JR (1988). Mechanism of fluorescence concentration quenching of carboxyfluorescein in liposomes: energy transfer to nonfluorescent dimers. Analytical Biochemistry.

[bib18] Cone CD, Cone CM (1976). Induction of mitosis in mature neurons in central nervous system by sustained depolarization. Science.

[bib19] de Silva AP, Gunaratne HQN, Habib-Jiwan J-L, McCoy CP, Rice TE, Soumillion J-P (1995). New fluorescent model compounds for the study of photoinduced electron transfer: the influence of a molecular electric field in the excited state. Angewandte Chemie International Edition in English.

[bib20] Deal PE, Kulkarni RU, Al-Abdullatif SH, Miller EW (2016). Isomerically pure tetramethylrhodamine voltage reporters. Journal of the American Chemical Society.

[bib21] Dumas D, Stoltz JF (2005). New tool to monitor membrane potential by FRET voltage sensitive dye (FRET-VSD) Using spectral and fluorescence lifetime imaging microscopy (FLIM). Interest in cell engineering. Clinical Hemorheology and Microcirculation.

[bib22] Edelstein AD, Tsuchida MA, Amodaj N, Pinkard H, Vale RD, Stuurman N (2014). Advanced methods of microscope control using μManager software. Journal of Biological Methods.

[bib23] Enderlein J, Erdmann R (1997). Fast fitting of multi-exponential decay curves. Optics Communications.

[bib24] Gao L, Liang J, Li C, Wang LV (2014). Single-shot compressed ultrafast photography at one hundred billion frames per second. Nature.

[bib25] González JE, Tsien RY (1997). Improved indicators of cell membrane potential that use fluorescence resonance energy transfer. Chemistry & Biology.

[bib26] Gordon JA (1991). Use of vanadate as protein-phosphotyrosine phosphatase inhibitor. Methods in Enzymology.

[bib27] Grenier V, Daws BR, Liu P, Miller EW (2019). Spying on neuronal membrane potential with genetically targetable voltage indicators. Journal of the American Chemical Society.

[bib28] Gross E, Bedlack RS, Loew LM (1994). Dual-wavelength ratiometric fluorescence measurement of the membrane dipole potential. Biophysical Journal.

[bib29] Harvey CD, Yasuda R, Zhong H, Svoboda K (2008). The spread of ras activity triggered by activation of a single dendritic spine. Science.

[bib30] Hodgkin AL, Katz B (1949). The effect of sodium ions on the electrical activity of giant axon of the squid. The Journal of Physiology.

[bib31] Horn R, Korn SJ (1992). Prevention of rundown in electrophysiological recording. Methods in Enzymology.

[bib32] Hou JH, Venkatachalam V, Cohen AE (2014). Temporal dynamics of microbial rhodopsin fluorescence reports absolute membrane voltage. Biophysical Journal.

[bib33] Huang CJ, Harootunian A, Maher MP, Quan C, Raj CD, McCormack K, Numann R, Negulescu PA, González JE (2006). Characterization of voltage-gated sodium-channel blockers by electrical stimulation and fluorescence detection of membrane potential. Nature Biotechnology.

[bib34] Huang YL, Walker AS, Miller EW (2015). A photostable silicon rhodamine platform for optical voltage sensing. Journal of the American Chemical Society.

[bib35] Huang X, Jan LY (2014). Targeting potassium channels in cancer. The Journal of Cell Biology.

[bib36] Lakowicz JR, Szmacinski H, Johnson ML (1992). Calcium imaging using fluorescence lifetimes and long-wavelength probes. Journal of Fluorescence.

[bib37] Lee SJ, Escobedo-Lozoya Y, Szatmari EM, Yasuda R (2009). Activation of CaMKII in single dendritic spines during long-term potentiation. Nature.

[bib38] Levin M (2014). Molecular bioelectricity: how endogenous voltage potentials control cell behavior and instruct pattern regulation in vivo. Molecular Biology of the Cell.

[bib39] Levitt JA, Kuimova MK, Yahioglu G, Chung P-H, Suhling K, Phillips D (2009). Membrane-bound molecular rotors measure viscosity in live cells via fluorescence lifetime imaging. The Journal of Physical Chemistry C.

[bib40] Li LS (2007). Fluorescence probes for membrane potentials based on mesoscopic electron transfer. Nano Letters.

[bib41] Liu M, Jia M, Pan H, Li L, Chang M, Ren H, Argoul F, Zhang S, Xu J (2014). Instrument response standard in time-resolved fluorescence spectroscopy at visible wavelength: quenched fluorescein sodium. Applied Spectroscopy.

[bib42] Liu P, Grenier V, Hong W, Muller VR, Miller EW (2017). Fluorogenic targeting of voltage-sensitive dyes to neurons. Journal of the American Chemical Society.

[bib43] Loew LM, Scully S, Simpson L, Waggoner AS (1979). Evidence for a charge-shift electrochromic mechanism in a probe of membrane potential. Nature.

[bib44] Lückhoff A, Clapham DE (1994). Calcium channels activated by depletion of internal calcium stores in A431 cells. Biophysical Journal.

[bib45] Magde D, Rojas GE, Seybold PG (1999). Solvent dependence of the fluorescence lifetimes of xanthene dyes. Photochemistry and Photobiology.

[bib46] Magni M, Meldolesi J, Pandiella A (1991). Ionic events induced by epidermal growth factor: evidence that hyperpolarization and stimulated cation influx play a role in the stimulation of cell growth. The Journal of Biological Chemistry.

[bib47] Maher MP, Wu NT, Ao H (2007). pH-Insensitive FRET voltage dyes. Journal of Biomolecular Screening.

[bib48] Malinow R, Tsien RW (1990). Presynaptic enhancement shown by whole-cell recordings of long-term potentiation in hippocampal slices. Nature.

[bib49] Marquèze-Pouey B, Mailfert S, Rouger V, Goaillard JM, Marguet D (2014). Physiological epidermal growth factor concentrations activate high affinity receptors to elicit calcium oscillations. PLOS ONE.

[bib50] McKeithan WL, Savchenko A, Yu MS, Cerignoli F, Bruyneel AAN, Price JH, Colas AR, Miller EW, Cashman JR, Mercola M (2017). An automated platform for assessment of congenital and drug-induced arrhythmia with hiPSC-derived cardiomyocytes. Frontiers in Physiology.

[bib51] Meşe G, Richard G, White TW (2007). Gap junctions: basic structure and function. The Journal of Investigative Dermatology.

[bib52] Miller EW, Lin JY, Frady EP, Steinbach PA, Kristan WB, Tsien RY (2012). Optically monitoring voltage in neurons by photo-induced electron transfer through molecular wires. PNAS.

[bib53] Montana V, Farkas DL, Loew LM (1989). Dual-wavelength ratiometric fluorescence measurements of membrane potential. Biochemistry.

[bib54] Moolenaar WH, Yarden Y, de Laat SW, Schlessinger J (1982). Epidermal growth factor induces electrically silent Na+ influx in human fibroblasts. The Journal of Biological Chemistry.

[bib55] Moolenaar WH, Aerts RJ, Tertoolen LG, de Laat SW (1986). The epidermal growth factor-induced calcium signal in A431 cells. The Journal of Biological Chemistry.

[bib56] Mozhayeva GN, Naumov AP, Kuryshev YA (1989). Epidermal growth factor activates calcium-permeable channels in A 431 cells. Biochimica Et Biophysica Acta (BBA) - Molecular Cell Research.

[bib57] Ouadid-Ahidouch H, Le Bourhis X, Roudbaraki M, Toillon RA, Delcourt P, Prevarskaya N (2001). Changes in the K+ current-density of MCF-7 cells during progression through the cell cycle: possible involvement of a h-Ether.a-Gogo K+ channel.. Receptors & Channels.

[bib58] Pandiella A, Malgaroli A, Meldolesi J, Vicentini LM (1987). EGF raises cytosolic Ca2+ in A431 and Swiss 3T3 cells by a dual mechanism. Redistribution from intracellular stores and stimulated influx. Experimental Cell Research.

[bib59] Pandiella A, Magni M, Lovisolo D, Meldolesi J (1989). The effects of epidermal growth factor on membrane potential. J. Biol. Chem.

[bib60] Peppelenbosch MP, Tertoolen LGJ, De Laat SW (1991). Epidermal growth factor-activated calcium and potassium channels. J. Biol. Chem.

[bib61] Peterka DS, Takahashi H, Yuste R (2011). Imaging voltage in neurons. Neuron.

[bib62] Raspe M, Kedziora KM, van den Broek B, Zhao Q, de Jong S, Herz J, Mastop M, Goedhart J, Gadella TWJ, Young IT (2015). SiFLIM: single-image frequency-domain FLIM provides fast and photon-efficient lifetime data. Methods.

[bib63] Reddy RJ, Gajadhar AS, Swenson EJ, Rothenberg DA, Curran TG, White FM (2016). Early signaling dynamics of the epidermal growth factor receptor. PNAS.

[bib64] Ross WN, Reichardt LF (1979). Species-specific effects on the optical signals of voltage-sensitive dyes. The Journal of Membrane Biology.

[bib65] Rothenberg P, Reuss L, Glaser L (1982). Serum and epidermal growth factor transiently depolarize quiescent BSC-1 epithelial cells. PNAS.

[bib66] Schindelin J, Arganda-Carreras I, Frise E, Kaynig V, Longair M, Pietzsch T, Preibisch S, Rueden C, Saalfeld S, Schmid B, Tinevez JY, White DJ, Hartenstein V, Eliceiri K, Tomancak P, Cardona A (2012). Fiji: an open-source platform for biological-image analysis. Nature Methods.

[bib67] Thul PJ, Åkesson L, Wiking M, Mahdessian D, Geladaki A, Ait Blal H, Alm T, Asplund A, Björk L, Breckels LM, Bäckström A, Danielsson F, Fagerberg L, Fall J, Gatto L, Gnann C, Hober S, Hjelmare M, Johansson F, Lee S, Lindskog C, Mulder J, Mulvey CM, Nilsson P, Oksvold P, Rockberg J, Schutten R, Schwenk JM, Sivertsson Å, Sjöstedt E, Skogs M, Stadler C, Sullivan DP, Tegel H, Winsnes C, Zhang C, Zwahlen M, Mardinoglu A, Pontén F, von Feilitzen K, Lilley KS, Uhlén M, Lundberg E (2017). A subcellular map of the human proteome. Science.

[bib68] Tsuchiya W, Okada Y (1982). Membrane potential changes associated with differentiation of enterocytes in the rat intestinal villi in culture. Developmental Biology.

[bib69] Vallat R (2018). Pingouin: statistics in python. Journal of Open Source Software.

[bib70] Wang L (2012). Measurements and implications of the membrane dipole potential. Annual Review of Biochemistry.

[bib71] Williams SR, Mitchell SJ (2008). Direct measurement of somatic voltage clamp errors in central neurons. Nature Neuroscience.

[bib72] Wonderlin WF, Woodfork KA, Strobl JS (1995). Changes in membrane potential during the progression of MCF-7 human mammary tumor cells through the cell cycle. Journal of Cellular Physiology.

[bib73] Woodford CR, Frady EP, Smith RS, Morey B, Canzi G, Palida SF, Araneda RC, Kristan WB, Kubiak CP, Miller EW, Tsien RY (2015). Improved PeT molecules for optically sensing voltage in neurons. Journal of the American Chemical Society.

[bib74] Wulff H, Miller MJ, Hansel W, Grissmer S, Cahalan MD, Chandy KG (2000). Design of a potent and selective inhibitor of the intermediate-conductance Ca2+-activated K+ channel, IKCa1: a potential immunosuppressant. PNAS.

[bib75] Yang M, Brackenbury WJ (2013). Membrane potential and cancer progression. Frontiers in Physiology.

[bib76] Yellen G, Mongeon R (2015). Quantitative two-photon imaging of fluorescent biosensors. Current Opinion in Chemical Biology.

[bib77] Zhang J, Davidson RM, Wei MD, Loew LM (1998). Membrane electric properties by combined patch clamp and fluorescence ratio imaging in single neurons. Biophysical Journal.

[bib78] Zhang H, Reichert E, Cohen AE (2016). Optical electrophysiology for probing function and pharmacology of voltage-gated ion channels. eLife.

[bib79] Zheng K, Bard L, Reynolds JP, King C, Jensen TP, Gourine AV, Rusakov DA (2015). Time-resolved imaging reveals heterogeneous landscapes of nanomolar Ca2+ in neurons and astroglia. Neuron.

[bib80] Zhou Y, Wong CO, Cho KJ, van der Hoeven D, Liang H, Thakur DP, Luo J, Babic M, Zinsmaier KE, Zhu MX, Hu H, Venkatachalam K, Hancock JF (2015). Membrane potential modulates plasma membrane phospholipid dynamics and K-Ras signaling. Science.

