# Supplementary material for: Optical estimation of absolute membrane potential using fluorescence lifetime imaging
Source: eLife. 2019 Sep 23;8:e44522. doi: 10.7554/eLife.44522 (PMC6814365; doi:10.7554/eLife.44522)
Supplement: Figure 1—source data 1. — aMeasurements vary too much to be converted to absolute voltage or interpreted across populations of cells. This variability is attributable to numerous confounding factors, including dye loading, photobleaching, and sample movement (Peterka et al., 2011). bWhile in principle less variable than a single-color fluorescence intensity measurement, in practice, the signal depends strongly on the loading of two independent lipophilic indicators (Adams and Levin, 2012; Maher et al., 2007), which can vary substantially. cANEPPS excitation ratios depend on a variety of non-voltage factors, in particular the membrane composition, leading to substantial artifacts in optical Vmem determinations (Zhang et al., 1998; Gross et al., 1994). dWith the GEVI CAESR in our hands, apparently poor protein trafficking produces large amounts of non-voltage-sensitive signal, which contaminates the FLIM recording and contributes to high cell to cell variability (Figure 1—figure supplement 4, Materials and methods). ePatch-clamp electrophysiology requires physical contact with the cell of interest, which causes damage to the cell and, in whole cell configurations, washout of intracellular factors. Slight movement of the cell or sample generally result in loss of the patch. fMovement of the cell and photobleaching of the dye both cause large changes to the signal over seconds to minutes. gRatio-calibrated imaging approaches use a second signal (usually another color of fluorescence) to correct for differences in dye concentration or changes in the region of interest that contaminate single-color intensity signals. If the rate of photobleaching is the same for both components, photobleaching artifacts can also be avoided. hLimited by photon count rates. iLimited by probe movement in the membrane, which depends mostly on lipophilicity (Briggman et al., 2010). jPhoton counting based lifetime imaging, like epifluorescence intensity imaging, is limited by photon count rates. Large numbers of photons [file elife-44522-fig1-data1.docx]

### Fig. 1, Source Data 1. Comparison of available approaches for measuring membrane potential in cells.

|  | **Patch-clamp electro-**  **physiology** | **Single color fluorescence intensity imaging** | **Two component ratiometric fluorescent sensors (FRET-oxonol)** | **Ratiometric, electrochromic fluorescent sensors (ANEPPS)** | **GEVI-based FLIM approaches** | **FLIM with VoltageFluors (VF-FLIM, *this work*)** |
| --- | --- | --- | --- | --- | --- | --- |
| **Absolute V_mem_ resolution (between cell comparisons)** | excellent | none^a^ | very poor^b^ | poor^c^ | very poor^d^ | good |
| **Quantification of V_mem_ changes on a given cell** | excellent | none^a^ | poor^b^ | poor^c^ | poor^d^ | excellent |
| **Compatibility with long time scales** | poor^e^ | poor^f^ | good^g^ | good^g^ | good | good |
| **Temporal resolution** | sub-millisecond | ~1 ms^h^ | 2-500 ms^i^ | ~1 ms^h^ | seconds^j^ | seconds^j^ |
| **Minimal invasiveness, damage** | very poor^e^ | good | poor^k^ | good | good | good |
| **Throughput (cells/day)** | 10s | 1000s | 1000s | 1000s | 1000s | 1000s |
| **Spatial resolution** | Single value per electrode^l^ | Subcellular | Subcellular | Subcellular | Single value per laser path^m^ | Single cell^n^ |

^a^Measurements vary too much to be converted to absolute voltage or interpreted across populations of cells. This variability is attributable to numerous confounding factors, including dye loading, photobleaching, and sample movement ^9^.

^b^While in principle less variable than a single-color fluorescence intensity measurement, in practice, the signal depends strongly on the loading of two independent lipophilic indicators ^13,80^, which can vary substantially.

^c^ANEPPS excitation ratios depend on a variety of non-voltage factors, in particular the membrane composition, leading to substantial artifacts in optical V_mem_ determinations ^16,17^.

^d^With the GEVI CAESR in our hands, apparently poor protein trafficking produces large amounts of non-voltage-sensitive signal, which contaminates the FLIM recording and contributes to high cell to cell variability ([Fig. 1-supplement 6](#_Fig._1,_S5.)**,** [Methods](#_Resolution_of_VF-FLIM)).

^e^Patch-clamp electrophysiology requires physical contact with the cell of interest, which causes damage to the cell and, in whole cell configurations, washout of intracellular factors. Slight movement of the cell or sample generally result in loss of the patch.

^f^Movement of the cell and photobleaching of the dye both cause large changes to the signal over seconds to minutes.

^g^Ratio-calibrated imaging approaches use a second signal (usually another color of fluorescence) to correct for differences in dye concentration or changes in the region of interest that contaminate single-color intensity signals. If the rate of photobleaching is the same for both components, photobleaching artifacts can also be avoided.

^h^Limited by photon count rates.

^i^Limited by probe movement in the membrane, which depends mostly on lipophilicity ^12^.

^j^Photon counting based lifetime imaging, like epifluorescence intensity imaging, is limited by photon count rates. Large numbers of photons per pixel must be collected to fit TCSPC FLIM data, often using a line scanning confocal approach, leading to slower acquisition speeds than epifluorescence-based intensity imaging.

^k^Toxicity from capacitive load of the sensor ^12^.

^l^The spatial resolution of electrophysiology is compromised by space clamp error, preventing interpretation of V_mem_ in regions far from the electrode (e.g. many neuronal processes) ^35,36^.

^m^As demonstrated by Cohen and co-workers ^27^; in our hands with CAESR, we also experienced significant improvements in voltage resolution by fitting a single curve per FLIM image instead of processing the images pixel-wise (see [Methods](#_Analysis_of_CAESR))

^n^In this work, we calibrated VF-FLIM for V_mem_ measurements with single cell resolution. In principle, subcellular spatial resolution could be achieved with the VF-FLIM technique.
