# Supplementary material for: Optical estimation of absolute membrane potential using fluorescence lifetime imaging
Source: eLife. 2019 Sep 23;8:e44522. doi: 10.7554/eLife.44522 (PMC6814365; doi:10.7554/eLife.44522)
Supplement: Figure 1—source data 2. — Fluorescein and erythrosin B standards were measured in drops of solution placed on a coverslip. For VF dyes, voltage sensitivities from intensity-based fluorescence imaging in HEK293T cells (%ΔF/F, percent change in fluorescence intensity for a voltage step from −60 mV to +40 mV) are from previously published work (Woodford et al., 2015). Lifetime data were obtained from voltage-clamp electrophysiology of HEK293T cells loaded with 100 nM VF. Lifetime listed here is the average 0 mV lifetime from the electrophysiology calibration. % Δτ/τ is the percent change in lifetime corresponding to a 100 mV step from −60 mV to +40 mV. Lifetime sample sizes: fluorescein 25, erythrosin B 25, VF2.1.Cl 17, VF2.0.Cl 17. For lifetime standards, each measurement was taken on a separate day. VF2.1.Cl data in HEK293T is duplicated in Figure 2—source data 1. Values are tabulated as mean ± SEM. [file elife-44522-fig1-data2.docx]

**Fig. 1, Source Data 2**. Properties of lifetime standards and VoltageFluor dyes.

|  | %ΔF/F | %Δτ/τ | Lifetime (ns) |
| --- | --- | --- | --- |
| Fluorescein | N/A | N/A | 4.008 ± 0.009 |
| Erythrosin B | N/A | N/A | 0.083 ± 0.001 |
| VF2.1.Cl | 27 | 22.4 ± 0.4% | 1.77 ± 0.02 |
| VF2.0.Cl | 0 | 0.11 ± 0.05% | 3.482 ± 0.004 |

**Fig. 1, Source Data 2.** Properties of lifetime standards and VoltageFluor dyes. Fluorescein and erythrosin B standards were measured in drops of solution placed on a coverslip. For VF dyes, voltage sensitivities from intensity-based fluorescence imaging in HEK293T cells (%ΔF/F, percent change in fluorescence intensity for a voltage step from -60 mV to +40 mV) are from previously published work ^30^. Lifetime data were obtained from voltage-clamp electrophysiology of HEK293T cells loaded with 100 nM VF. Lifetime listed here is the average 0 mV lifetime from the electrophysiology calibration. % Δτ/τ is the percent change in lifetime corresponding to a 100 mV step from -60 mV to +40 mV. Lifetime sample sizes: fluorescein 25, erythrosin B 25, VF2.1.Cl 17, VF2.0.Cl 17. For lifetime standards, each measurement was taken on a separate day. VF2.1.Cl data in HEK293T is duplicated in Figure 2 – source data 1. Values are tabulated as mean ± SEM.
