# Supplementary material for: Optical estimation of absolute membrane potential using fluorescence lifetime imaging
Source: eLife. 2019 Sep 23;8:e44522. doi: 10.7554/eLife.44522 (PMC6814365; doi:10.7554/eLife.44522)
Supplement: Figure 1—source data 3. — Data are compiled from Figure 1 (VF-FLIM, this work), Figure 1—figure supplement 4 (CAESR; Brinks et al., 2015), and Figure 1—figure supplement 5 (Di-8-ANEPPS; Zhang et al., 1998). All data were obtained by simultaneous whole cell voltage clamp electrophysiology and optical recording in HEK293T (VF-FLIM n = 17 cells, CAESR n = 9, di-8-ANEPPS n = 16). Calculation of intra and inter cell accuracies are performed via root-mean-square deviation (RMSD) and discussed in detail in the Methods (see Resolution of VF-FLIM…). Regions of interest were chosen at the plasma membrane in all cases. Di-8-ANEPPS data are presented as the ratio of signal obtained with blue excitation to signal obtained with green excitation (R, see Materials and methods) and are not normalized to the 0 mV R. [file elife-44522-fig1-data3.docx]

**Fig. 1, Source Data 3.** Comparison of optical approaches to absolute V_mem_ determination in HEK293T cells.

| **Strategy** | **Sensitivity** | **0 mV value** | **Intra-cell RMSD** | **Inter-cell RMSD** |
| --- | --- | --- | --- | --- |
| VF2.1.Cl fluorescence lifetime | 3.50 ± 0.08 ps/mV | 1.77 ± 0.02 ns | 3.5 ± 0.4 mV | 19 mV |
| CAESR fluorescence lifetime | -1.2 ± 0.1 ps/mV | 2.0 ± 0.2 ns | 33 ± 7 mV | 370 mV |
| di-8-ANEPPS excitation ratio | 0.0039 ± 0.0004 R/mV | 1.8 ± 0.2 | 18 ± 3 mV | 150 mV |

**Fig. 1, Source Data 3.** Comparison of optical approaches to absolute V_mem_ determination in HEK293T cells. Data are compiled from **Fig. 1** (VF-FLIM, this work), **Fig. 1 – supplement 4** (CAESR ^27^), and **Fig. 1 – supplement 5** (Di-8-ANEPPS ^17^). All data were obtained by simultaneous whole cell voltage clamp electrophysiology and optical recording in HEK293T (VF-FLIM n=17 cells, CAESR n=9, di-8-ANEPPS n=16). Calculation of intra and inter cell accuracies are performed via root-mean-square deviation (RMSD) and discussed in detail in the Methods (see **Resolution of VF-FLIM…**). Regions of interest were chosen at the plasma membrane in all cases. Di-8-ANEPPS data are presented as the ratio of signal obtained with blue excitation to signal obtained with green excitation (R, see **Methods**) and are not normalized to the 0 mV R.
