# Supplementary material for: Optical estimation of absolute membrane potential using fluorescence lifetime imaging
Source: eLife. 2019 Sep 23;8:e44522. doi: 10.7554/eLife.44522 (PMC6814365; doi:10.7554/eLife.44522)
Supplement: Figure 2—source data 1. — Whole-cell voltage-clamp electrophysiology was used to determine the relationship between VF2.1.Cl lifetime and membrane potential in five different cell lines. Parameters of this linear model are listed above. The %Δτ/τ is the percent change in the lifetime observed for a voltage step from −60 mV to +40 mV. The intra-cell RMSD represents the accuracy for quantifying voltage changes in a particular cell (see Materials and methods). The inter-cell RMSD represents the expected variability in single-trial absolute Vmem determinations. Sample sizes: A431 12, CHO 8, HEK293T 17, MCF-7 24, MDA-MB-231 11. All values are tabulated as mean ± SEM. [file elife-44522-fig2-data1.docx]

**Fig. 2, Source Data 1**. Lifetime-V_mem_ standard curves for VF2.1.Cl lifetime in various cell lines.

| **Cell Line** | **Slope (ps/mV)** | **0 mV lifetime (ns)** | **%Δτ/τ** | **RMSD, intra-cell (mV)** | **RMSD, inter-cell (mV)** |
| --- | --- | --- | --- | --- | --- |
| **A431** | 3.55 ± 0.08 | 1.74 ± 0.02 | 23.3 ± 0.4% | 3.8 ± 0.7 | 15 |
| **CHO** | 3.68 ± 0.25 | 1.74 ± 0.01 | 24 ± 2% | 5 ± 1 | 10 |
| **HEK293T** | 3.50 ± 0.08 | 1.77 ± 0.02 | 22.4 ± 0.4% | 3.5 ± 0.4 | 19 |
| **MCF-7** | 3.07 ± 0.03 | 1.87 ± 0.01 | 18.2 ± 0.2% | 3.4 ± 0.3 | 23 |
| **MDA-MB-231** | 3.47 ± 0.06 | 1.86 ± 0.02 | 21.1 ± 0.4% | 3.0 ± 0.4 | 16 |

**Fig. 2, Source Data 1.** Lifetime-V_mem_ standard curves for VF2.1.Cl lifetime in various cell lines. Whole-cell voltage-clamp electrophysiology was used to determine the relationship between VF2.1.Cl lifetime and membrane potential in five different cell lines. Parameters of this linear model are listed above. The %Δτ/τ is the percent change in the lifetime observed for a voltage step from -60 mV to +40 mV. The intra-cell RMSD represents the accuracy for quantifying voltage changes in a particular cell (see Methods). The inter-cell RMSD represents the expected variability in single-trial absolute V_mem_ determinations. Sample sizes: A431 12, CHO 8, HEK293T 17, MCF-7 24, MDA-MB-231 11. All values are tabulated as mean ± SEM.
