# Supplementary material for: Optical estimation of absolute membrane potential using fluorescence lifetime imaging
Source: eLife. 2019 Sep 23;8:e44522. doi: 10.7554/eLife.44522 (PMC6814365; doi:10.7554/eLife.44522)
Supplement: Figure 3—source data 1. — Comparison of optically-determined resting membrane potential values (in millivolts) and previously reported values. This table summarizes data presented in Figure 3 and Figure 3—figure supplement 1. Optically determined membrane potentials were calculated from lifetime-Vmem standard curves (Figure 2—source data 1). For tabulated literature values, measures of error and central tendency were used from the original publication. In some cases, none were given or only ranges were discussed. The mean of the reported ephys values is the mean of the values listed here. Sample sizes for resting and elevated K+, respectively: A431 1056, 368; CHO 2410, 1310; HEK293T 1613, 520; MCF-7 1259, 681; MDA-MB-231 1840, 558. [file elife-44522-fig3-data1.docx]

**Fig. 3, Source Data 1.** V_mem_ measurements made with VF-FLIM agree with previously reported values.

|  | VF-FLIM | | Patch-clamp electrophysiology | |
| --- | --- | --- | --- | --- |
| Cell Line | Resting V_mem_ (mean ± SEM) | High K^+^ V_mem_ (mean ± SEM) | Compiled reported average or median V_mem_ in cells at rest | Mean of ephys. values |
| A431 | -41 ± 5 | -26 ± 5 | -64 ± 1 (mean ± SEM)^1^ | -64 |
| CHO | -53 ± 4 | -20 ± 4 | -21 ± 2 (mean ± SEM, 4 cells)^2^  -31 ± 2.6 (mean ± SEM)^3^  -35 (ranging -10 to -65 mV)^4^ | -30 |
| HEK293T | -47 ± 5 | -6 ± 5 | -45 (ranging -40 to -50 mV)^5^  -52 ± 1 (mean ± SEM)^6^  -35 ± 2 (mean ± SEM)^7^ | -44 |
| MCF-7 | -29 ± 5 | 4 ± 5 | -23 ± 1 (median ± SE of median)^8^  -36 ± 5 (mean ± SEM)^9^  -41 ± 20 (mean ± SD)^10^  -42 (no error given)^11^  -42 ± 5 (mean ± SEM)^12^ | -36 |
| MDA-MB-231 | -38 ± 5 | -15 ± 5 | -19 ± 3 (mean ± SEM)^13^  -26 ± 8 (mean ± SEM)^14^  -39 ± 5 (mean ± SEM)^9^ | -28 |

**Fig. 3, Source Data 1.** V_mem_ measurements made with VF-FLIM agree with previously reported values. Comparison of optically-determined resting membrane potential values (in millivolts) and previously reported values. This table summarizes data presented in Fig. 3 and Fig. 3 – supplement 1. Optically determined membrane potentials were calculated from lifetime-V_mem_ standard curves (Fig. 2 – source data 1). For tabulated literature values, measures of error and central tendency were used from the original publication. In some cases, none were given or only ranges were discussed. The mean of the reported ephys values is the mean of the values listed here. Sample sizes for resting and elevated K^+^, respectively: A431 1056, 368; CHO 2410, 1310; HEK293T 1613, 520; MCF-7 1259, 681; MDA-MB-231 1840, 558.

**References for Fig. 3 – Source Data 1**

(1) Moolenaar, W. H.; Aerts, R. J.; Tertoolen, L. G. J.; De Laat, S. W. The Epidermal Growth Factor-Induced Calcium Signal in A431 Cells. *J. Biol. Chem.* **1986**, *261* (1), 279–284.

(2) Defarias, F. P.; Stevens, S. P.; Leonard, R. J. Stable Expression of Human Kv1.3 Potassium Channels Resets the Resting Membrane Potential of Cultured Mammalian Cells. *Receptors Channels* **1995**, *3* (4), 273–281.

(3) Walker, B. D.; Valenzuela, S. M.; Singleton, C. B.; Tie, H.; Bursill, J. A.; Wyse, K. R.; Qiu, M. R.; Breit, S. N.; Campbell, T. J. Inhibition of HERG Channels Stably Expressed in a Mammalian Cell Line by the Antianginal Agent Perhexiline Maleate. *Br. J. Pharmacol.* **1999**, *127* (1), 243–251. https://doi.org/10.1038/sj.bjp.0702502.

(4) Cone, C. D.; Tongier, M. Contact Inhibition of Division: Involvement of the Electrical Transmembrane Potential. *J. Cell. Physiol.* **1973**, *82* (3), 373.

(5) Fliegert, R.; Glassmeier, G.; Schmid, F.; Cornils, K.; Genisyuerek, S.; Harneit, A.; Schwarz, J. R.; Guse, A. H. Modulation of Ca2+ Entry and Plasma Membrane Potential by Human TRPM4b. *FEBS J.* **2007**, *274* (3), 704–713. https://doi.org/10.1111/j.1742-4658.2006.05614.x.

(6) Babai, N.; Kanevsky, N.; Dascal, N.; Rozanski, G. J.; Singh, D. P.; Fatma, N.; Thoreson, W. B. Anion-Sensitive Regions of L-Type CaV1.2 Calcium Channels Expressed in HEK293 Cells. *PLoS One* **2010**, *5* (1). https://doi.org/10.1371/journal.pone.0008602.

(7) Hsu, K.; Han, J.; Shinlapawittayatorn, K.; Deschenes, I.; Marbán, E. Membrane Potential Depolarization as a Triggering Mechanism for Vpu-Mediated HIV-1 Release. *Biophys. J.* **2010**, *99* (6), 1718–1725. https://doi.org/10.1016/j.bpj.2010.07.027.

(8) Wonderlin, W. F.; Woodfork, K. A.; Strobl, J. S. Changes in Membrane Potential during the Progression of MCF-7 Human Mammary Tumor Cells through the Cell Cycle. *J. Cell. Physiol.* **1995**, *165* (1), 177–185. https://doi.org/10.1002/jcp.1041650121.

(9) Berzingi, S.; Newman, M.; Yu, H.-G. Altering Bioelectricity on Inhibition of Human Breast Cancer Cells. *Cancer Cell Int.* **2016**, *16*, 72. https://doi.org/10.1186/s12935-016-0348-8.

(10) Ouadid-Ahidouch, H.; Le Bourhis, X.; Roudbaraki, M.; Toillon, R. A.; Delcourt, P.; Prevarskaya, N. Changes in the K+ Current-Density of MCF-7 Cells during Progression through the Cell Cycle: Possible Involvement of a h-Ether.a-Gogo K+ Channel. *Receptors Channels* **2001**, *7* (5), 345–356. https://doi.org/10.1111/j.1464-410X.2009.08500.x.

(11) Wang, S. Y.; Melkoumian, Z.; Woodfork, K. A.; Cather, C.; Davidson, A. G.; Wonderlin, W. F.; Strobl, J. S. Evidence for an Early G1 Ionic Event Necessary for Cell Cycle Progression and Survival in the MCF-7 Human Breast Carcinoma Cell Line. *J. Cell. Physiol.* **1998**, *176* (3), 456–464. https://doi.org/10.1002/(sici)1097-4652(199809)176:3<456::aid-jcp2>3.0.co;2-n.

(12) Marino, A. A.; Iliev, I. G.; Schwalke, M. A.; Gonzalez, E.; Marler, K. C.; Flanagan, C. A. Association between Cell Membrane Potential and Breast Cancer. *Tumor Biol.* **1994**, *15* (2), 82–89. https://doi.org/10.1159/000217878.

(13) Hammadi, M.; Chopin, V.; Matifat, F.; Dhennin-Duthille, I.; Chasseraud, M.; Sevestre, H.; Ouadid-Ahidouch, H. Human Ether À-Gogo K+ Channel 1 (HEag1) Regulates MDA-MB-231 Breast Cancer Cell Migration through Orai1-Dependent Calcium Entry. *J. Cell. Physiol.* **2012**, *227* (12), 3837–3846. https://doi.org/10.1002/jcp.24095.

(14) Thurber, A. E.; Nelson, M.; Frost, C. L.; Levin, M.; Brackenbury, W. J.; Kaplan, D. L. IK Channel Activation Increases Tumor Growth and Induces Differential Behavioral Responses in Two Breast Epithelial Cell Lines. *Oncotarget* **2017**, *8* (26), 42382–42397. https://doi.org/10.18632/oncotarget.16389.
